# Assessment of safety and intranasal neutralizing antibodies of HPMC-based human anti-SARS-CoV-2 IgG1 nasal spray in healthy volunteers

**DOI:** 10.1038/s41598-023-42539-7

**Published:** 2023-09-20

**Authors:** Thanarath Imsuwansri, Thitinan Jongthitinon, Niramon Pojdoung, Nuntana Meesiripan, Siriwan Sakarin, Chatikorn Boonkrai, Tossapon Wongtangprasert, Tanapati Phakham, Thittaya Audomsun, Chadaporn Attakitbancha, Pijitra Saelao, Phijitra Muanwien, Maoxin Tim Tian, Songsak Tongchusak, Bhrus Sangruji, Dhammika Leshan Wannigama, Chenphop Sawangmake, Watchareewan Rodprasert, Quynh Dang Le, Steven Dwi Purbantoro, Kananuch Vasuntrarak, Sirirat Nantavisai, Supakit Sirilak, Ballang Uppapong, Sompong Sapsutthipas, Sakalin Trisiriwanich, Thitiporn Somporn, Asmah Usoo, Natthakarn Mingngamsup, Supaporn Phumiamorn, Porawan Aumklad, Kwanputtha Arunprasert, Prasopchai Patrojanasophon, Praneet Opanasopit, Norapath Pesirikan, Ladda Nitisaporn, Jesada Pitchayakorn, Thana Narkthong, Bancha Mahong, Kumchol Chaiyo, Kanjana Srisutthisamphan, Ratchanont Viriyakitkosol, Songklot Aeumjaturapat, Anan Jongkaewwattana, Sakarn Bunnag, Trairak Pisitkun

**Affiliations:** 1 Department of Medical Services, National Cancer Institute, Ministry of Public Health, Bangkok, Thailand; 2 Faculty of Medicine, Center of Excellence in Systems Biology, Chulalongkorn University, Bangkok, Thailand; 3 The Excellence Chulalongkorn Comprehensive Cancer Center, King Chulalongkorn Memorial Hospital, Bangkok, Thailand; 4 School of Arts and Sciences, Tufts University, Massachusetts, USA; 5 Department of Microbiology, Faculty of Medicine, Chulalongkorn University, Bangkok, Thailand; 6 School of Medicine, Faculty of Health and Medical Sciences, The University of Western Australia, Nedlands, Western Australia Australia; 7 Department of Pharmacology, Faculty of Veterinary Science, Chulalongkorn University, Bangkok, Thailand; 8 Faculty of Veterinary Science, Veterinary Stem Cell and Bioengineering Innovation Center (VSCBIC), Chulalongkorn University, Bangkok, Thailand; 9 Veterinary Stem Cell and Bioengineering Research Unit, Faculty of Veterinary Science, Chulalongkorn University, Bangkok, Thailand; 10 Academic Affairs, Faculty of Veterinary Science, Chulalongkorn University, Bangkok, Thailand; 11 Department of Medical Sciences, Ministry of Public Health, Nonthaburi, Thailand; 12 Department of Medical Sciences, Institute of Biological Products, Ministry of Public Health, Nonthaburi, Thailand; 13 Faculty of Pharmacy, Silpakorn University, Nakhon Pathom, Thailand; 14 The Government Pharmaceutical Organization, Bangkok, Thailand; 15 Virology and Cell Technology Research Team, National Center for Genetic Engineering and Biotechnology (BIOTEC), National Science and Technology Development Agency (NSTDA), Pathumthani, Thailand; 16 Otolaryngology Department, King Chulalongkorn Memorial Hospital, Bangkok, Thailand

**Keywords:** Biotechnology, Biologics, Translational research

## Abstract

An HPMC-based nasal spray solution containing human IgG1 antibodies against SARS-CoV-2 (nasal antibody spray or NAS) was developed to strengthen COVID-19 management. NAS exhibited potent broadly neutralizing activities against SARS-CoV-2 with PVNT_50_ values ranging from 0.0035 to 3.1997 μg/ml for the following variants of concern (ranked from lowest to highest): Alpha, Beta, Gamma, ancestral, Delta, Omicron BA.1, BA.2, BA.4/5, and BA.2.75. Biocompatibility assessment showed no potential biological risks. Intranasal NAS administration in rats showed no circulatory presence of human IgG1 anti-SARS-CoV-2 antibodies within 120 h. A double-blind, randomized, placebo-controlled trial (NCT05358873) was conducted on 36 healthy volunteers who received either NAS or a normal saline nasal spray. Safety of the thrice-daily intranasal administration for 7 days was assessed using nasal sinuscopy, adverse event recording, and self-reporting questionnaires. NAS was well tolerated, with no significant adverse effects during the 14 days of the study. The SARS-CoV-2 neutralizing antibodies were detected based on the signal inhibition percent (SIP) in nasal fluids pre- and post-administration using a SARS-CoV-2 surrogate virus neutralization test. SIP values in nasal fluids collected immediately or 6 h after NAS application were significantly increased from baseline for all three variants tested, including ancestral, Delta, and Omicron BA.2. In conclusion, NAS was safe for intranasal use in humans to increase neutralizing antibodies in nasal fluids that lasted at least 6 h.

## Introduction

The enduring waves of SARS-CoV-2 breakthrough infections create a global impediment that requires additional measures beyond vaccination to mitigate this perpetual situation. SARS-CoV-2 is an RNA virus with the characteristic of multiple spike glycoproteins on its envelope^1^. Through airborne transmission, the nasopharyngeal epithelium is SARS-CoV-2’s primary portal, which incubates the virus to a high viral load for shedding and dissemination^2,3^. The receptor-binding domain (RBD) on SARS-CoV-2 spike glycoproteins specifically binds angiotensin-converting enzyme 2 (ACE2) expressed on the plasma membrane of target cells, setting off a cell entry cascade of the virus^4^. The local defense system at the nasopharyngeal mucosa, especially via antibody-mediated immunity that rapidly interferes with RBD-ACE2 engagement, is thus regarded as the genuine instrument for COVID-19 prevention^5–7^. However, after systemic vaccination, the neutralizing antibody levels in the nasopharyngeal mucosa naturally decline and are typically insufficient to prevent SARS-CoV-2 breakthrough infections in the long term. However, it is impractical to repeatedly boost systemic vaccines to maintain the protective level of mucosal immunity at all times. Therefore, an innovative approach is needed for this unprecedented situation. Recently, strategies to bolster mucosal immunity using an active or passive route via intranasal administration of vaccines or antibodies, respectively, have gained critical momentum^5,8–12^.

**Figure 1:**
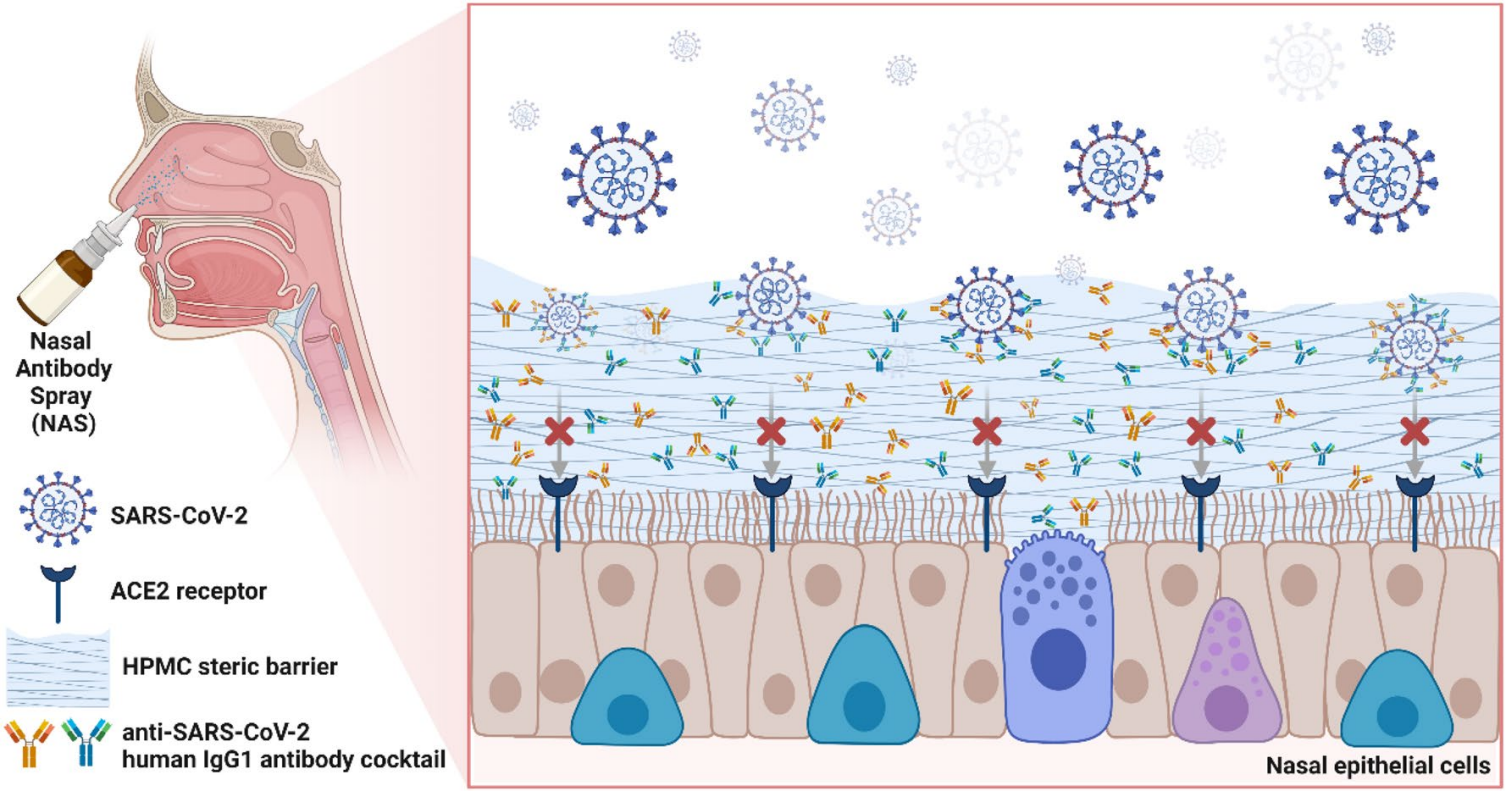
Mechanism of action of NAS. NAS provides the dual-action physical barrier on nasal mucosa by (1) forming of steric barrier at the cell surface by HPMC and (2) inhibiting SARS-CoV-2 viral particles via an anti-SARS-CoV-2 human IgG1 antibody cocktail. This figure was created with BioRender.com.

Nasal Antibody Spray (NAS) is an HPMC-based nasal spray solution containing human IgG1 antibodies. It has been approved by the Thai FDA as an innovative medical device platform (Class 4) to support mucosal immunity against SARS-CoV-2 infections via a dual mechanism of action through antibody-mediated specific inhibition coupled with a steric barrier (Fig. 1). Human IgG1 antibodies included in the NAS platform are monoclonal antibodies with potent broadly neutralizing activities against SARS-CoV-2 screened from elite responders who have fully recovered from COVID-19^13^. NAS is strategically formulated to allow a timely modification of the antibody component to react to the potential immune escape of future SARS-CoV-2 variants. In addition to human IgG1 antibodies, a mucoadhesive cellulose derivative, hypromellose (HPMC), is another key ingredient of NAS that forms a steric barrier on nasopharyngeal mucosa to prevent SARS-CoV-2 from entering target cells. This study aims to evaluate NAS’s pseudovirus neutralization potencies, biocompatibility, and safety and intranasal neutralizing antibodies in healthy volunteers in accordance with the ICH-GCP guidelines. This study was approved by the Ethics Committee of the Department of Medical Services, Ministry of Public Health, Thailand (Approval No. 0001/2565, approval date: 12/04/2022) and registered with the ClinicalTrials.gov; Date of first registration: 03/05/2022; Registration number: NCT05358873.

## Results

### Pseudovirus neutralization potencies

Pseudovirus microneutralization assays were performed as previously described^14,15^ to assess the neutralization potency of NAS against various SARS-CoV-2 pseudoviruses, i.e., ancestral, Alpha, Delta, Omicron BA.1, BA.2, A.4/5, and BA.2.75. The results revealed that NAS potently neutralized all mentioned pseudoviruses with 50% pseudovirus neutralization titers (PVNT_50_) ranging from 0.0035 to 3.1997 µg/ml (Table 1). It should be noted that the magnitude of neutralization against Omicron BA.2.75, which is among the most immune-evasive SARS-CoV-2 variants tested, appears to be less potent than that of other variants. However, the concentration of the antibody cocktail in NAS is still 78.13-fold higher than Omicron BA.2.75’s PVNT_50_.

**Table 1:** Pseudovirus neutralization potencies of NAS.

SARS-CoV-2 variants	PVNT_50_ (µg/ml)
Ancestral	0.0092
Alpha	0.0035
Beta	0.0044
Gamma	0.0055
Delta	0.0117
Omicron BA.1	0.0219
Omicron BA.2	0.0135
Omicron BA.4/5	0.1919
Omicron BA.2.75	3.1997

### Biocompatibility assessment

**Figure 2:**
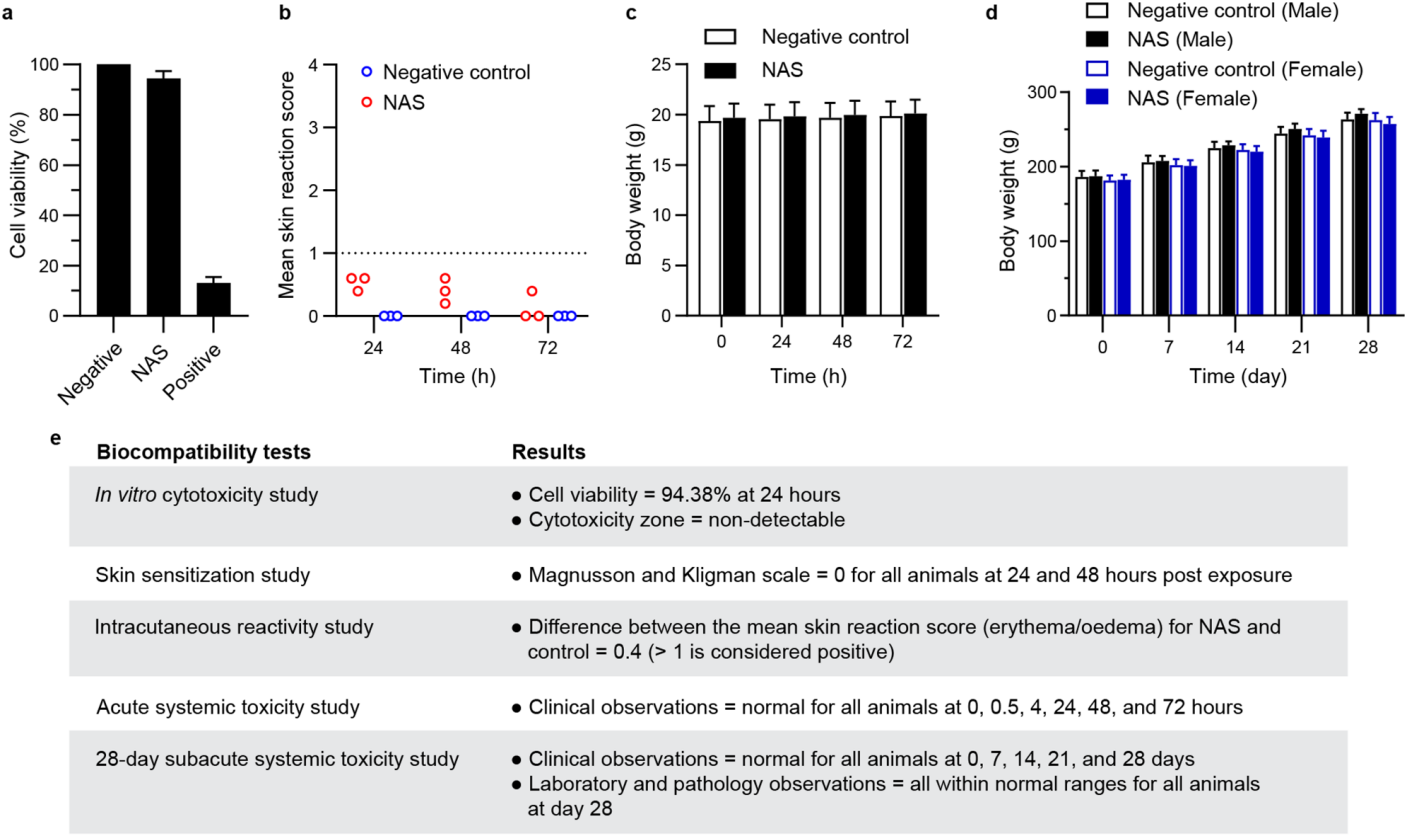
Biocompatibility study of NAS. **(a)** In vitro cytotoxicity of NAS was assessed using the direct contact method in Balb/c 3T3 cells. Cell viability percentage after 24 h of co-culture was determined. Negative (HDPE) and positive (natural rubber latex) controls were included. **(b)** Intracutaneous reactivity test in New Zealand white rabbits was used to evaluate the mean skin reaction scores (erythema/oedema) at different time points. The samples were intracutaneously injected (0.2 ml) at five test sites per treatment, and skin reactions were visually scored at 24, 48, and 72 h post-injection. Physiological saline was used as a negative control. **(c)** Swiss albino mice were orally treated with NAS at 50 ml/kg body weight to assess acute systemic toxicity. Body weight and clinical signs of toxicity were monitored. Physiological saline was used as a negative control. **(d)** Wistar rats were orally treated with NAS at 10 ml/kg body weight to evaluate subacute (28-day) systemic toxicity. Body weight and any clinical signs of toxicity were monitored, and hematology, clinical biochemistry, and pathology tests were performed at the end of the study. **(e)** Key results of the biocompatibility study of NAS are summarized.

A biocompatibility assessment was performed, which included several in vitro and in vivo studies (Fig. 2). First, an in vitro cytotoxicity study was conducted using the Balb/c 3T3 cell line by the direct contact method. The results demonstrated that NAS was non-cytotoxic to the cells, with 94.38% cell viability observed at 24 h post-coincubation, as shown in Fig. 2a. A skin sensitization study was also conducted in guinea pigs, which revealed that NAS was considered a non-sensitizer (Magnusson and Kligman scale = 0 for all animals at all time points); please see Table S4 in Supplemental Data S1 for more details. Additionally, an irritation study was conducted in New Zealand white rabbits using an intracutaneous reactivity test. The results showed that NAS was deemed non-reactive in rabbits, with mean reaction scores (erythema/oedema) below 1.0 (Fig. 2b). An acute systemic toxicity study was conducted via oral administration of NAS at 50 ml/kg body weight in Swiss albino mice, which demonstrated no systemic toxicity. Furthermore, a gradual increase in body weight was observed in all the animals at the end of the experiment (Fig. 2c). Lastly, a 28-day subacute systemic toxicity study was conducted via oral administration of NAS at 10 ml/kg body weight in rats, which showed no mortality/morbidity or any other clinical signs of toxicity during the study period. Rat body weights were monitored and are displayed in Fig. 2d. Hematology, urinalysis, clinical biochemistry, and pathology results were within normal ranges (Supplemental Data S1). All key biocompatibility results are summarized in Fig. 2e. These findings demonstrate that NAS is safe and does not induce any signs of toxicity in animals during the study period.

### Circulatory levels of human IgG1 anti-SARS-CoV-2 antibodies after intranasal application of NAS

In order to assess the potential systemic effects of intranasal application of NAS, quantitative measurement of human IgG1 anti-SARS-CoV-2 antibodies after intranasal application of NAS was determined in rats. A single dose of NAS (20 µg/kg) was intranasally applied to 10-week-old female Sprague–Dawley rats (n=13). This dose was calculated based on the intended single-use amount in humans (2 µg/kg) multiplied by a human-to-rat conversion factor of ten^16^. Three rats without any intervention were used as controls. Rat serum was collected at 1, 4, 8, 24, 48, 72, 96, and 120 h post-administration. Human IgG1 anti-SARS-CoV-2 antibodies were not detected by ELISA in the bloodstream of rats at any time point during the 120 h post-intranasal administration (limit of quantitation = 0.165 ng/ml, see Supplemental Data S2).

### Safety of NAS application

**Figure 3:**
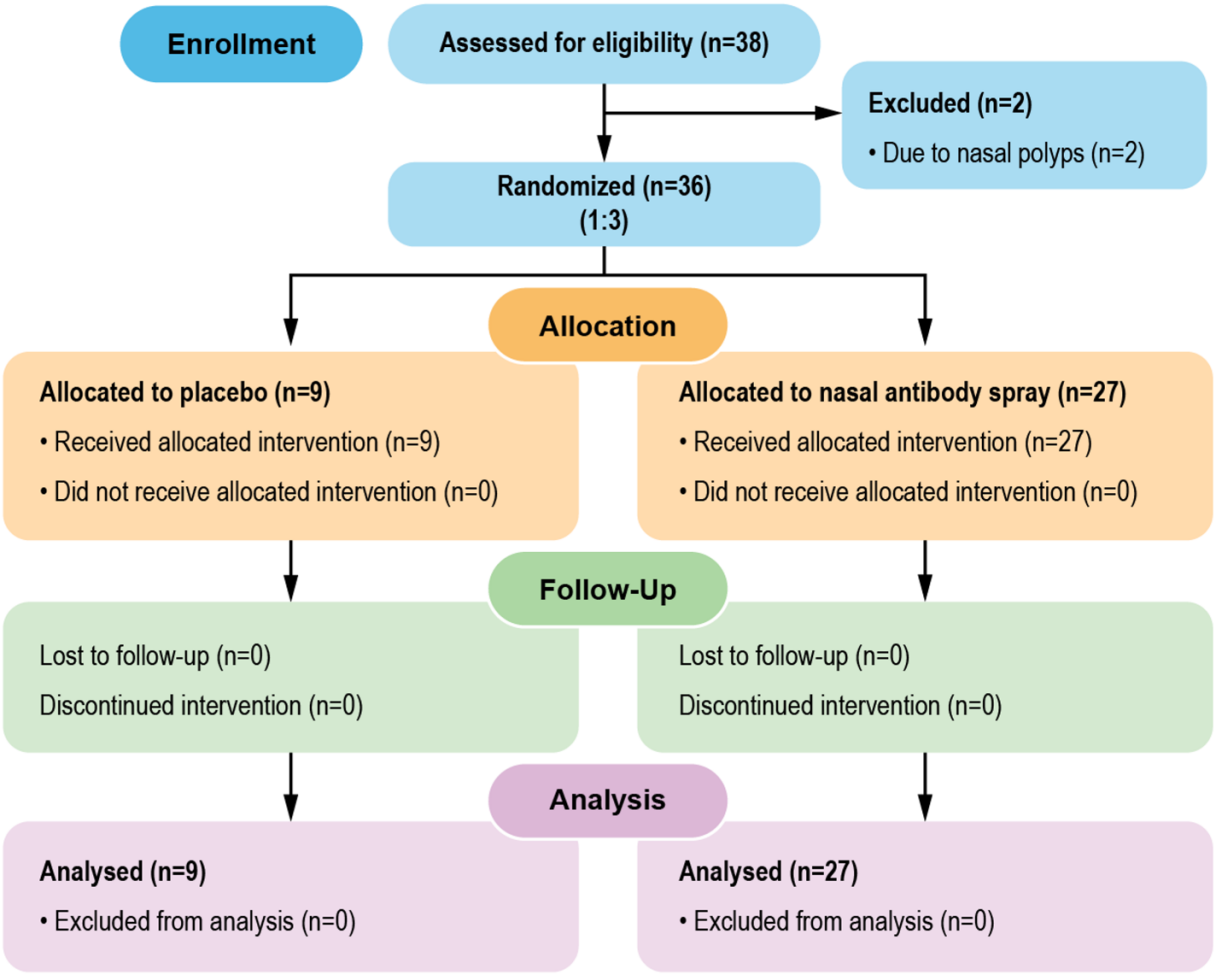
Study flow chart. Thirty-eight healthy volunteers were enrolled to evaluate the safety and detect intranasal neutralizing antibodies before and after a placebo or NAS application. Thirty-six volunteers were randomized into a 1:3 ratio to receive a placebo (n=9) and NAS (n=27), while the other 2 were excluded due to nasal polyps. Safety was assessed using sinuscopy, adverse event recording, and self-reporting questionnaires. SARS-CoV-2 neutralizing antibodies were detected in the nasal fluids taken from volunteers pre- and post-administration.

**Figure 4:**
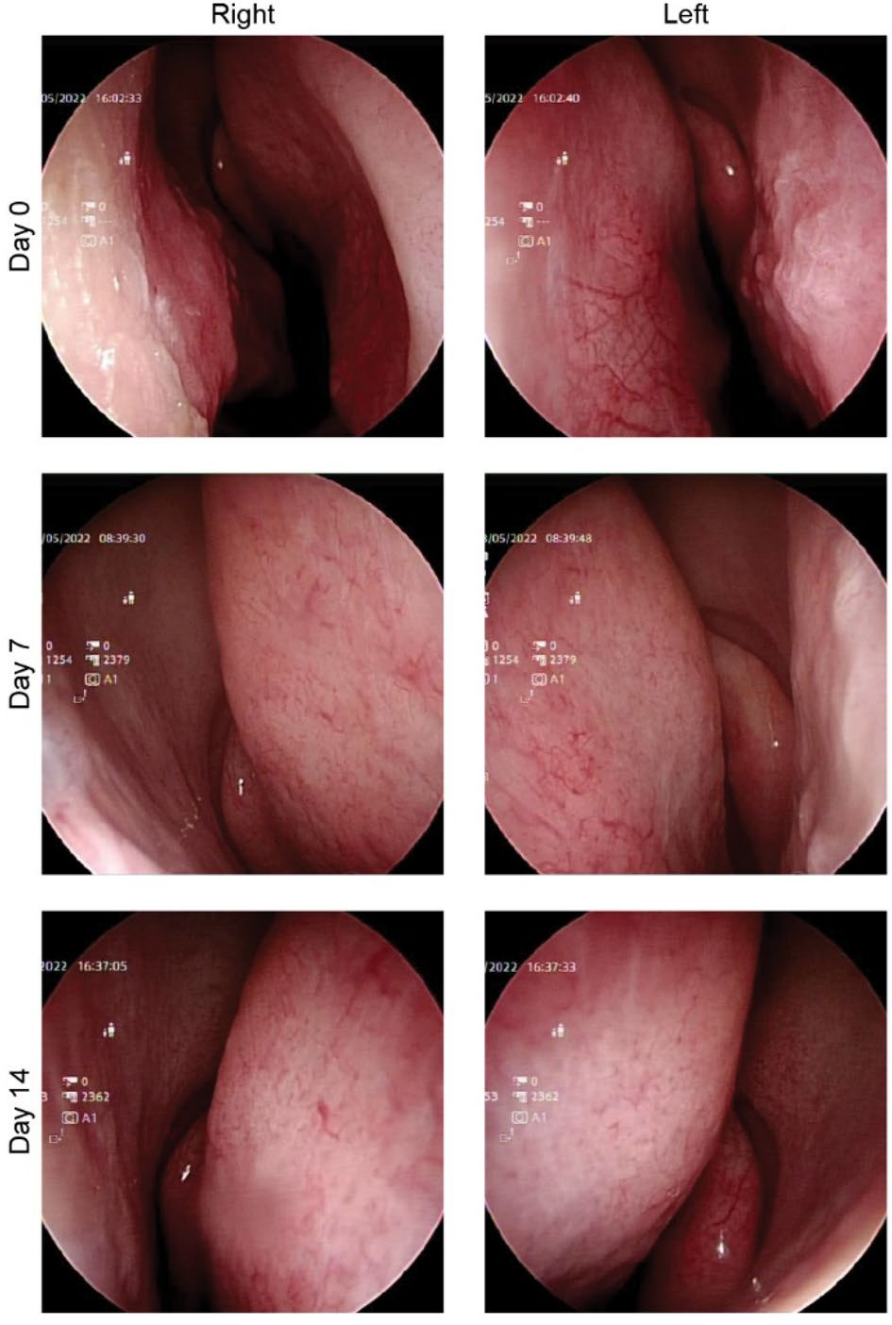
Representative nasal sinuscopy images after NAS application. The participants’ nasal sinus in both nostrils was imaged using a sinuscope on days 0, 7, and 14. No inflammation signs or any appearance changes were observed.

To evaluate the safety and tolerability of NAS, 38 healthy volunteers were enrolled (Fig. 3 shows a flow chart of this study). However, two of them were excluded due to nasal polyps. Thirty-six participants were randomly assigned in a 1:3 ratio to receive a placebo or NAS. The characteristics of all participants enrolled in the study are summarized in Table 2. We used objective (nasal sinuscopy) and subjective (SNOT-22 and TNSS questionnaires) assessments. Participants sprayed 2 puffs of the study products into each nostril thrice daily at 8 am, 2 pm, and 8 pm for 7 days and were then followed up for another 7 days. Nasal sinuscopy was performed on all participants on days 0, 7, and 14. Nasal sinuscopy findings were evaluated using the modified Lund–Kennedy endoscopic scoring system. Following this scoring system, we did not find any changes in nasal mucosa appearance or any signs of inflammation in either the NAS or placebo group at any given time point (Fig. 4, Table 3). Supplemental Data S3 shows nasal sinuscopy images displayed in random order of participants.

**Table 2:** Baseline characteristics of participants in the study.

Characteristics	Placebo (n=9)	NAS (n=27)	Total (n=36)
Age, median (range)	30 (24–45)	32 (24–45)	32 (24–45)
Gender; n (%)			
Male	6 (66.66)	12 (44.44)	18 (50.00)
Female	3 (33.33)	15 (55.57)	18 (50.00)
Average height, cm	166.56	165.41	165.69
Average weight, kg	67.29	63.36	64.34
Average BMI, kg/m^2^	24.05	23.06	23.31
Average blood pressure (systolic/ diastolic), mmHg	127.33/73.22	121.65/72.12	123.11/72.40
Average heart rate, beats per minute	79.56	81.69	81.14
Average respiration rate, breath per minute	19.22	18.80	18.91
Average spO_2_, %	99	98.5	98.75
Received at least 2 shots of COVID-19 vaccines, %	100	100	100

**Table 3:** Safety assessment via nasal sinuscopy.

Physical examination via nasal sinuscopy	Placebo (n=9)			NAS (n=27)		
Day	0	7	14	0	7	14
Total modified Lund–Kennedy endoscopic score (mean ± SE)	0 ± 0	0 ± 0	0 ± 0	0 ± 0	0 ± 0	0 ± 0
Two-tailed t-test	Not statistically significant					

SNOT-22 and TNSS questionnaires were used to evaluate nasal symptoms throughout the 2 weeks of the study period. These questionnaires have been validated for patient-reported outcomes of chronic rhinosinusitis, allergic rhinitis, and other sinonasal outcomes^17,18^. For both questionnaires, each participant scored the severity of each symptom daily. Fourteen-day accumulative severity scores for each symptom were compared between the NAS and placebo groups. The results showed that for both questionnaires, the highest reported score was 0 (no problem or none) for every symptom in both groups. For some symptoms, especially runny nose/rhinorrhea, a score of 1 (SNOT-22: a very mild problem and TNSS: mild) was reported at a low percentage in the NAS group; however, these mild nasal symptoms were recovered without any medical treatments in all cases. The runny nose symptom is likely a result of the slightly viscous HPMC-based solution of NAS. HPMC helps extend the retention time of the antibodies in the nasal cavity by reducing mucociliary clearance and might explain this nasal symptom. Overall, both groups had no substantial difference in sinonasal symptoms (Tables 4, 5).

**Table 4:** Self-reported symptoms by SNOT-22 questionnaire (14-day accumulative events).

SNOT-22 score	% Of each severity level												Adjusted *P* value
	Placebo (n=9)						NAS (n=27)						
	0	1	2	3	4	5	0	1	2	3	4	5	
Need to blow nose	100.0	0.0	0.0	0.0	0.0	0.0	97.9	2.1	0.0	0.0	0.0	0.0	> 0.9999
Nasal blockage	100.0	0.0	0.0	0.0	0.0	0.0	97.9	2.1	0.0	0.0	0.0	0.0	> 0.9999
Sneezing	100.0	0.0	0.0	0.0	0.0	0.0	99.2	0.8	0.0	0.0	0.0	0.0	> 0.9999
Runny nose	99.2	0.8	0.0	0.0	0.0	0.0	93.4	6.3	0.3	0.0	0.0	0.0	< 0.0001
Cough	100.0	0.0	0.0	0.0	0.0	0.0	98.1	1.9	0.0	0.0	0.0	0.0	> 0.9999
Post-nasal discharge	97.6	2.4	0.0	0.0	0.0	0.0	97.4	2.6	0.0	0.0	0.0	0.0	> 0.9999
Thick nasal discharge	100.0	0.0	0.0	0.0	0.0	0.0	99.2	0.8	0.0	0.0	0.0	0.0	> 0.9999
Ear fullness	100.0	0.0	0.0	0.0	0.0	0.0	99.7	0.3	0.0	0.0	0.0	0.0	> 0.9999
Dizziness	100.0	0.0	0.0	0.0	0.0	0.0	100.0	0.0	0.0	0.0	0.0	0.0	> 0.9999
Ear pain	100.0	0.0	0.0	0.0	0.0	0.0	100.0	0.0	0.0	0.0	0.0	0.0	> 0.9999
Facial pain/pressure	100.0	0.0	0.0	0.0	0.0	0.0	100.0	0.0	0.0	0.0	0.0	0.0	> 0.9999
Decreased sense of smell/taste	100.0	0.0	0.0	0.0	0.0	0.0	99.7	0.3	0.0	0.0	0.0	0.0	> 0.9999
Difficulty falling asleep	100.0	0.0	0.0	0.0	0.0	0.0	99.7	0.3	0.0	0.0	0.0	0.0	> 0.9999
Wake up at night	100.0	0.0	0.0	0.0	0.0	0.0	100.0	0.0	0.0	0.0	0.0	0.0	> 0.9999
Lack of a good night’s sleep	100.0	0.0	0.0	0.0	0.0	0.0	99.7	0.3	0.0	0.0	0.0	0.0	> 0.9999
Wake up tired	100.0	0.0	0.0	0.0	0.0	0.0	100.0	0.0	0.0	0.0	0.0	0.0	> 0.9999
Fatigue	100.0	0.0	0.0	0.0	0.0	0.0	100.0	0.0	0.0	0.0	0.0	0.0	> 0.9999
Reduced productivity	100.0	0.0	0.0	0.0	0.0	0.0	100.0	0.0	0.0	0.0	0.0	0.0	> 0.9999
Reduced concentration	100.0	0.0	0.0	0.0	0.0	0.0	100.0	0.0	0.0	0.0	0.0	0.0	> 0.9999
Frustrated/restless/irritable	100.0	0.0	0.0	0.0	0.0	0.0	100.0	0.0	0.0	0.0	0.0	0.0	> 0.9999
Sad	100.0	0.0	0.0	0.0	0.0	0.0	100.0	0.0	0.0	0.0	0.0	0.0	> 0.9999
Embarrassed	100.0	0.0	0.0	0.0	0.0	0.0	100.0	0.0	0.0	0.0	0.0	0.0	> 0.9999

SNOT-22 score: 0 = No problem, 1 = Very mild problem, 2 = Mild or slight problem, 3 = Moderate problem, 4 = Severe problem, or 5 = Problem as bad as it can be.

**Table 5:** Self-reported symptoms by TNSS questionnaire (14-day accumulative events).

TNSS score	% Of each severity level								Adjusted *P* value
	Placebo (n=9)				NAS (n=27)				
	0	1	2	3	0	1	2	3	
Rhinorrhea	100.0	0.0	0.0	0.0	94.2	5.6	0.3	0.0	0.0005
Nasal congestion	100.0	0.0	0.0	0.0	98.7	1.3	0.0	0.0	> 0.9999
Nasal itching	100.0	0.0	0.0	0.0	98.9	1.1	0.0	0.0	> 0.9999
Sneezing	100.0	0.0	0.0	0.0	98.7	1.3	0.0	0.0	> 0.9999

TNSS score: 0 = None, 1 = Mild, 2 = Moderate, or 3 = Severe.

Additionally, treatment-emergent adverse events (TEAEs) were evaluated over 14 days of the study. No adverse events were reported from participants in either group (Table 6). Collectively, all assessments indicated that NAS was well tolerated, with no significant adverse effects in healthy volunteers.

**Table 6:** Self-reported treatment emergent adverse events (TEAEs).

Adverse events	No. of TEAEs	
	Placebo (n=9)	NAS (n=27)
Fatal (resulted in death)	0	0
A life-threatening occurrence	0	0
Requires inpatient hospitalization or prolongation of existing hospitalization	0	0
Results in persistent or significant disability/incapacity	0	0
Results in congenital anomaly/birth defect	N/A	N/A
A significant medical incident that, based upon appropriate medical judgment, may jeopardize the subject, and require medical or surgical intervention to prevent one of the outcomes listed above	0	0

### Detection of SARS-CoV-2 neutralizing antibodies in nasal fluids

**Figure 5:**
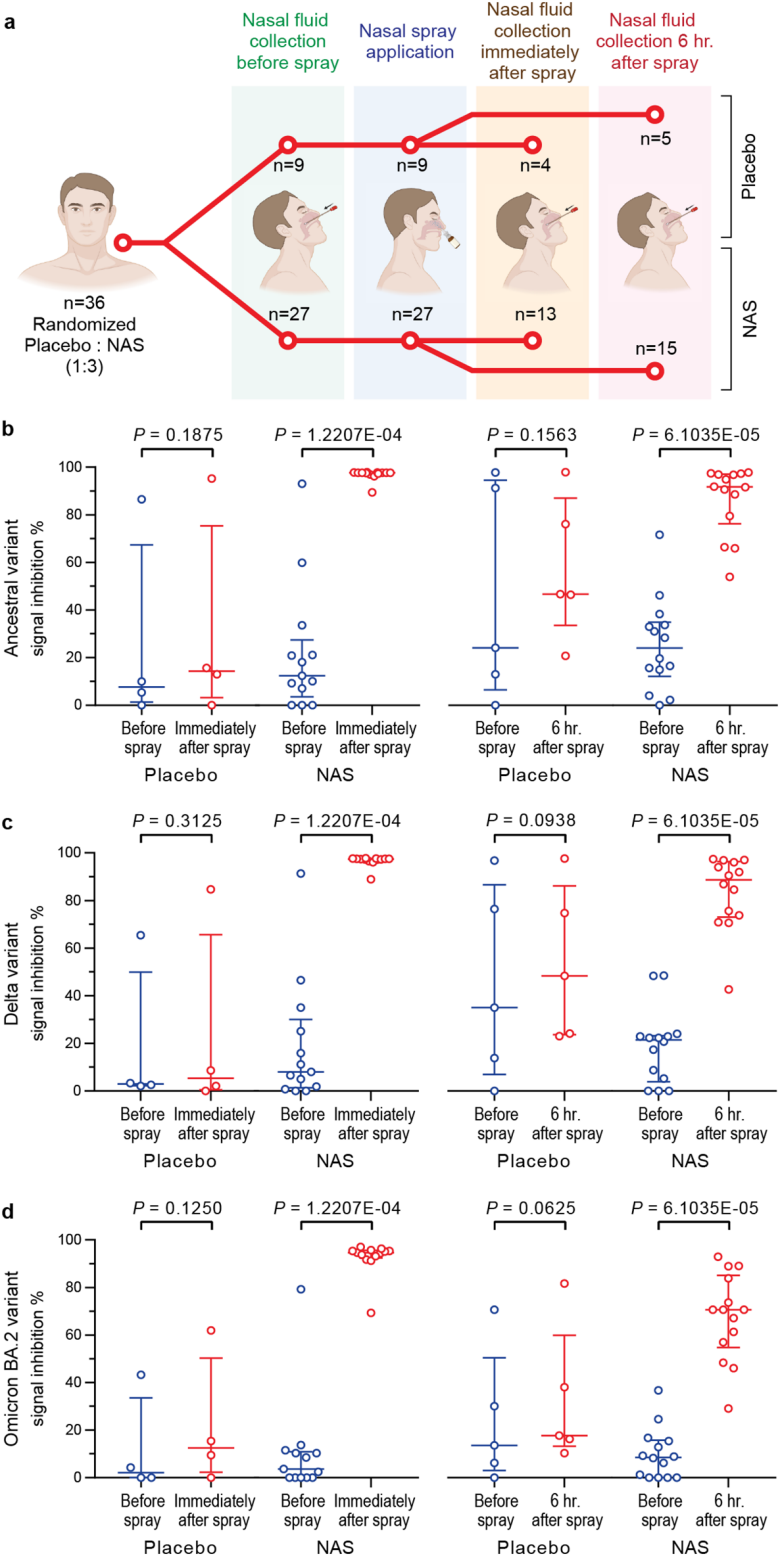
Assessment of SARS-CoV-2 neutralizing antibodies in nasal fluids before and after a placebo or NAS application. SARS-CoV-2 neutralizing antibodies in nasal fluids swabbed from pre- and post-product administration were detected using the cPass SARS-CoV-2 surrogate virus neutralization test. (**a**) Illustration of study design. SIP values against (**b**) ancestral, (**c**) Delta, and (**d**) Omicron BA.2 variants before and immediate or 6-h time point after a placebo or NAS application. Data are presented in IQR ± 25th–75th percentile.

Nasal fluid was collected by swabbing from both nostrils before and immediately or 6 h after the study product application in both the NAS and placebo groups (see Fig. 5a). A SARS-CoV-2 surrogate virus neutralization test (cPass, GenScript) was utilized to detect SARS-CoV-2 neutralizing antibodies in the collected nasal fluids.

The neutralizing antibodies against ancestral, Delta, or Omicron BA.2 RBD proteins were detected based on the signal inhibition percent (SIP) of the cPass assay. The SIP values against the ancestral variant in the nasal fluid from the NAS group were significantly increased from baseline at both time points (median of baseline vs. immediate time point: 12.41% vs. 97.58%; *P*-value = 1.2207E−4 and median of baseline vs. 6 h time point: 24.03% vs. 91.72%; *P*-value = 6.1035E−05), see Fig. 5b. Similarly, for the Delta and Omicron BA.2 variants (Fig. 5c,d), the nasal fluid from the NAS group showed a significant increase in the SIP values from the baseline at the immediate time point for both variants (median of baseline vs. immediate time point for Delta and Omicron BA.2 variants: 8.02% vs. 97.44%; *﻿P*-value = 1.2207E−4 and 3.64% vs. 94.65%; *﻿P*-value = 1.2207E−4). Likewise, the results at the 6-h time point still demonstrated a significant increase in the SIP values from baseline for both variants (median of baseline vs. 6-h time point for Delta and Omicron BA.2 variants: 21.53% vs. 88.67%; *P*-value = 6.1035E−05 and 8.59% vs. 70.60%; *P*-value = 6.1035E−05). In contrast, the nasal fluid at both time points from the placebo group did not show a significant difference in the SIP values from the baseline for all three variants tested. Supplemental Data S4 contains all detailed statistical reports from this evaluation.

## Discussion

NAS is a medical device innovated to support mucosal immunity against SARS-CoV-2 via a dual mechanism of action in which a potent broadly neutralizing human IgG1 anti-SARS-CoV-2 monoclonal antibody cocktail produces inhibitory effects against multiple variants of concern (VOCs) in nasal fluid, and a steric barrier-forming agent, HPMC, fortifies the mucus layer. NAS exhibited broadly neutralizing activities against SARS-CoV-2 pseudoviruses of ancestral, Alpha, Delta, Omicron BA.1, BA.2, BA.4/5, and BA.2.75 variants. To evaluate the safety and efficacy of NAS, preclinical studies were conducted following the ISO 10993 standards of medical devices. These studies showed good biocompatibility based on cytotoxicity, skin sensitization, and intracutaneous reactivity evaluations as well as satisfactory safety profiles by both acute and subacute systemic toxicity investigations. In animal studies, intranasal administration of NAS did not result in any detection of human IgG1 anti-SARS-CoV-2 antibodies in the bloodstream of rats at any time point during the 120 h of follow-up. This finding agrees with the knowledge that the nasal epithelial barrier only allows the passage of molecules smaller than 1000 Da^19^; thus, human IgG1’s molecular mass of 146,000 Da^20^ clearly prohibits the systemic distribution of the human IgG1 antibodies in NAS.

The randomized, placebo-controlled trial was conducted to assess the tolerability and effects of intranasal administration of NAS in 36 healthy participants. The trial revealed that NAS was well tolerated, without any changes in nasal mucosa appearance, any signs of inflammation, or any treatment-emergent adverse events for the entire 14 days of the study. Recently, the neutralizing antibody level in nasal fluids has been shown to correlate with the potential protective effects against Omicron infection^7,21,22^. Therefore, we detected the neutralizing antibodies in nasal fluids before and after NAS application in this study. We found a significant increase in neutralizing antibodies in nasal fluids after NAS application compared with baseline for all three variants tested, including ancestral, Delta, and Omicron BA.2. Immediately after NAS application, the SIP values in nasal fluids were increased to ≥ 91.69%. These intranasal effects remained significantly increased at 6 h after the application (≥ 70.60%).

The formulation of the NAS appears to be a significant factor contributing to its efficacy. This nasal spray uses HPMC, a compound commonly employed in pharmaceutical applications, especially as a viscosity enhancer that helps extend the retention time of the antibodies in the nasal cavity by reducing mucociliary clearance^23^, which in turn enhances the passive mucosal immunity. HPMC in the nasal spray formulation also demonstrated effectiveness in inhibiting SARS-CoV-2 infection by forming a gel matrix and reducing virus release by over 99.99% at a specific dose in cell culture^24^.

Nasal sprays with antibodies hold potential for protecting individuals at risk of virus exposure according to the classic susceptible-exposed-infectious-removed (SEIR) mathematical model^25^ and experimental reports^9,10,26^. The SEIR mathematical model demonstrates that intranasal administration of monoclonal antibodies provides initial protection to the mucosal surface, offering a valuable defense against viral infection^25^. However, to effectively control infections, sustained intranasal antibody prophylaxis would be required for a significant portion of the population. Additionally, post-exposure prophylaxis is crucial for reducing the development of severe disease and minimizing hospital admissions. In addition to NAS, other nasal sprays containing monoclonal antibodies have shown promise in preventing SARS-CoV-2 infection. One study evaluated a nasal spray formulation based on the monoclonal antibody 35B5, which provided 24-h effective protection against SARS-CoV-2 variants, including Alpha, Beta, Delta, and Omicron^10^. Another study engineered an IgM neutralizing antibody (IgM-14) that demonstrated prophylactic and therapeutic efficacy against SARS-CoV-2, including variants of concern^9^. Additionally, a nasal spray with an engineered human antibody exhibited the ability to block SARS-CoV-2 infection in both nasal and lung areas^26^. These findings highlight the potential of nasal sprays containing antibodies as preventive strategies against SARS-CoV-2, complementing the effectiveness of NAS.

While NAS shows promise in supporting mucosal immunity against SARS-CoV-2, it is important to acknowledge its limitations. Widespread and long-term administration of nasal sprays with antibodies poses logistical challenges. Additionally, relying solely on intranasal antibody prophylaxis cannot prevent the widespread transmission of the virus within a community. Therefore, NAS should be used in conjunction with other protective measures, such as non-pharmaceutical interventions, to fill the gap during the time needed to develop and manufacture effective vaccines. This approach can complement active immunization strategies and provide an additional layer of protection.

Collectively, NAS has shown safety and efficacy in increasing neutralizing antibodies in nasal fluids. However, to fully demonstrate the effectiveness of NAS in preventing COVID-19, a large-scale efficacy trial measuring COVID-19 incidence would be necessary. Further research and evaluation are needed to determine the potential role of NAS as a complementary tool in the fight against SARS-CoV-2.

## Methods

### Study product

Nasal Antibody Spray or NAS is an HPMC-based nasal spray solution containing human IgG1 anti-SARS-CoV-2 monoclonal antibodies, clones 1D1 and 3D2^13^ [U.S. provisional patent application No. US 63/248,115 and PCT/TH2022/000037], at 0.25 mg/ml. The concentration of the antibody cocktail in NAS was established at 833.33 times the 99% plaque reduction neutralization test (PRNT_99_) value against the SARS-CoV-2 delta variant.

### Pseudovirus-based neutralization assay

Lentiviral pseudoviruses containing SARS-CoV-2 spike were produced with slight modifications, as previously described by Di Genova et al.^27^. To generate pseudoviruses, a lentivirus backbone expressing a firefly luciferase reporter gene (pCSFLW), an expression plasmid expressing HIV-1 structural/regulatory proteins (pCMVR8.91), and pCAGGS expressing spike constructs were used. Unless otherwise noted, HEK293T/17 producing cells were seeded in 6-well plates 24 h before transfection with 600 ng pCMVR8.91, 600 ng pCSFLW, and 500 ng pCAGGS spike in OptiMEM containing 10 µl polyethylenimine (PEI). Transfected cells were incubated at 37 °C with 5% CO_2_. Twelve hours after transfection, the cells were washed and grown in DMEM containing 10% FBS. Pooled supernatants containing pseudoviruses were collected 72 h after transfection, centrifuged at 1500×*g* for 10 min at 4 °C to remove cell debris, aliquoted, and kept at – 80 °C.

To assess the neutralizing activities of Nasal Antibody Spray, a two-fold serial dilution of the NAS was conducted in a culture medium starting at a ratio of 1:40 (high-glucose DMEM without FBS). In a 96-well culture plate, the diluted samples were mixed with pseudoviruses bearing the SARS-CoV-2 spike of interest at a 1:1 v/v ratio. The input pseudovirus was adjusted to 1 × 10^5^ RLU per well. The antibody-pseudovirus mixture was then incubated at 37 °C for 1 h. Cell suspensions of HEK293T-ACE-2 cells pretransfected with pCAGGS expressing human TMPRSS2 (2 × 10^4^ cells/ml) were then seeded into each well of CulturPlate microplates (PerkinElmer). Finally, plates were incubated at 37 °C for 48 h, and neutralizing activities were detected by measuring luciferase activity, as previously described^28^.

### Biocompatibility testing

The biocompatibility of NAS was evaluated by the following 5 assessments conforming to the standards of medical devices (ISO 10993-5:2009, ISO 10993-10:2021, ISO 10993 Part 23: 2021, and ISO 10993-11:2017): (1) in vitro cytotoxicity using the direct contact method, (2) skin sensitization using the guinea pig maximization test, (3) intracutaneous reactivity potentials in New Zealand white rabbits, (4) acute systemic toxicity study via oral administration in mice, and (5) 28-day subacute systemic toxicity study via oral administration in rats (see details in Supplemental Methods). All animal experiments were performed in compliance with OECD Principles of Good Laboratory Practice ENV/MC/CHEM (98)17 (Revised 1997, issued January 1998) and applicable regulatory requirements including the US Food and Drug Administration’s GLP regulations, 21 CFR 58 (subparts B to G and J). The study is reported in accordance with ARRIVE guidelines (https://arriveguidelines.org).

### Enzyme-linked immunosorbent assay (ELISA)

ELISA was employed to quantify human IgG1 anti-SARS-CoV-2 antibody levels in the circulation after intranasal application of NAS in rats. In brief, rat serum samples diluted in 3% BSA in PBS buffer at a 1:10 dilution were added (100 µl/well) to an ELISA plate coated with 100 ng/well of Delta-variant RBD proteins (40592-V08H90, Sino Biological). Human IgG1 anti-SARS-CoV-2 antibodies were detected with goat anti-human IgG Fcγ-HRP antibody (109-005-098, Jackson Immuno Research) diluted 1:2,000 in 3% BSA in PBS buffer. The SIGMA*FAST* OPD (P9187, Sigma-Aldrich) substrate solution was used, and the reaction was stopped by adding 1 M H_2_SO_4_. The absorbance was measured at 492 nm by a Cytation 5 cell imaging multi-mode reader (BioTek).

### Clinical study design

This study was designed as a single-center, double-blind, randomized, placebo-controlled trial to evaluate the safety, tolerability, and clinical performance of NAS in healthy volunteers.

### Ethical considerations

This study was approved by the Ethics Committee of the Department of Medical Services, Ministry of Public Health, Thailand (Approval No. 0001/2565, approval date: 12/04/2022) and registered with the ClinicalTrials.gov; Date of first registration: 03/05/2022; Registration number: NCT05358873. All procedures were performed following the principles of the Declaration of Helsinki and the ICH-GCP guidelines. All participants provided written informed consent before the commencement of the study and voluntarily participated in this clinical trial.

### Participants

We calculated the sample size based on previous recommendations^29,30^ using Z statistics to assess the product’s safety, tolerability, and performance. We set a power of 69.0% to detect an effect size (E) of 0.5 with a threshold probability of rejecting the null hypothesis (α) of 5% (two-tailed). Furthermore, it was assumed that the data had a 20% probability of not rejecting the null hypothesis under the alternative hypothesis (β) and a standard deviation of change (SΔ) of the outcome of 1. The sample size (n) was calculated per the following formula.

$${\text{n}} = \left( {{\text{Z}}\alpha + {\text{Z}}\beta } \right) \times {\text{S}}\Delta /{\text{E}} = 31.39.$$

To ensure an adequate sample size in case participants dropped out, we added 4 more participants to attain the total number of 36 participants. Healthy volunteers interested in participating in the study were screened according to the inclusion and exclusion criteria described on ClinicalTrials.gov (NCT05358873).

All volunteers were randomly assigned in a 1:3 ratio into 2 groups: placebo (n = 9) and NAS (n = 27), as shown in Fig. 3.

### Product application

NAS and normal saline solution (placebo) were produced, packaged, and labeled with a double-blind, randomized code by the Government Pharmaceutical Organization (GPO), Ministry of Public Health, Thailand. Each participant was randomly assigned to receive either NAS or a placebo. Site staff gave participants instructions on the study product application, storage, and return. In brief, a trained nurse administered the study products into the nostrils of all participants for the first application to ensure that the correct dosage was delivered and that the application technique was consistent across all participants allowing for direct observation and immediate feedback to ensure proper administration by all participants. The participants were then instructed to self-administer the study products by spraying two puffs into their nostrils (100 µl per puff) thrice daily at 8 am, 2 pm, and 8 pm for 7 days. The training ensures that participants understand the correct technique, dosage, and frequency of administration. The volume and frequency of the study product application were based on reports of other nasal spray products containing anti-SARS-CoV-2 antibodies^26,31^. Any nasal products other than the study products were prohibited during the study period. To detect SARS-CoV-2 neutralizing antibodies in nasal fluids before and after the study product application in both the NAS and placebo groups, the nasal fluid collection was performed via nasal swabbing by two board-certified otorhinolaryngologists under a standardized procedure, i.e., swabbing was conducted on the entire accessible surface of the medial wall of the inferior turbinate in both the right and left nasal cavities, using two sterile cotton swabs, one for each nostril. Eight-hundred and fifty microliters of the sample dilution buffer (cPass SARS-CoV-2 neutralization antibody detection kit, GenScript) was used to elute nasal fluids from the head of swab sticks. To minimize the impact of nasal fluid collection on the safety evaluation, we control the number of nasal fluid collections after the study product application to only one time per participant by randomly assigning each participant to either an immediate or 6 h timepoint, as illustrated in Fig. 5a. No reapplication was performed after nasal fluid collection.

### Safety evaluation

Safety was assessed based on nasal sinuscopy examination, treatment-emergent adverse events (TEAEs), and Sino-Nasal Outcome Test-22 (SNOT-22) and Total Nasal Symptom Score (TNSS) questionnaires^17,32^. Nasal sinuscopy was performed on all participants using the Olympus ENF-V4 video rhinolaryngoscope on days 0, 7, and 14. Nasal sinuscopy findings were evaluated by an otorhinolaryngologist using the modified Lund-Kennedy endoscopic scoring system. Participants were asked to complete the SNOT-22 and TNSS questionnaires daily until the end of the study (Day 14).

### SARS-CoV-2 surrogate virus neutralization test

SARS-CoV-2 surrogate virus neutralization test in nasal fluid specimens against the ancestral, Delta, and Omicron BA.2 HRP-conjugated RBD proteins were determined using a SARS-CoV-2 surrogate virus neutralization test (cPass SARS-CoV-2 neutralization antibody detection kit, GenScript; A02087, Z03614, and Z03741). Nasal fluid specimens were diluted ~ tenfold in sample dilution buffer, and then the SARS-CoV-2 inhibitory effects were measured according to the instruction manual and reported as % inhibition against SARS-CoV-2.

### Statistical analysis

For the safety assessments of NAS, an unpaired two-tailed Mann–Whitney U test was used, and Dunn’s test was applied for multiple testing corrections. For the assessments of intranasal SARS-CoV-2 inhibitory effects of NAS, the difference in % inhibition before and after the study product applications was compared using a one-tailed Wilcoxon signed-rank test.

### Supplementary Information

Supplementary Information 1.

Supplementary Information 2.

Supplementary Information 3.

Supplementary Information 4.

Supplementary Information 5.

## Data Availability

All data generated or analyzed during this study are included in this published article.

## Abbreviations

HPMC: Hypromellose or hydroxypropyl methylcellulose
NAS: Nasal antibody spray
PVNT_50_: 50% Pseudovirus neutralization titer
SIP: Signal inhibition percent
