# Supplementary material for: Assessment of safety and intranasal neutralizing antibodies of HPMC-based human anti-SARS-CoV-2 IgG1 nasal spray in healthy volunteers
Source: Sci Rep. 2023 Sep 20;13:15648. doi: 10.1038/s41598-023-42539-7 (PMC10511465; doi:10.1038/s41598-023-42539-7)
Supplement: Supplementary file 1 — Supplementary Information 1. [file 41598_2023_42539_MOESM1_ESM.docx]

**Supplemental Data S1**

**Biocompatibility results**

Biocompatibility study was performed to evaluate the safety of the HPMC-based nasal spray solution containing human IgG1 antibodies against SARS-CoV-2 (Nasal Antibody Spray or NAS). The test item was NAS. Negative control was physiological saline solution or normal saline solution (NSS).

1. ***In vitro* cytotoxicity using the direct contact method**

Before treatment, approximately 80% confluency was confirmed in all the cultures. In the qualitative evaluation, the cells treated with the positive control showed a complete destruction of cell layers (grade 4). No such destruction was evident in the negative control. Quantitatively, the cells treated with the negative control had a mean viability of 100% and positive control had 13.48% viability. Based on qualitative and quantitative evaluations, the assay was considered as valid.

- **Qualitative evaluation**

The cultures treated with the negative control did not show any cytotoxic response (grade 0) whereas the positive control showed a severe positive cytotoxic response (grade 4). The assay was therefore considered valid. The cultures treated with NAS showed no detectable zone around or under specimen which was considered non-cytotoxic (grade 0) Table S1.

**Table S1 Qualitative evaluation**

| **Sample** | **Culture** | **Reactivity** | **Grade** |
| --- | --- | --- | --- |
| Negative Control  (HDPE) | 1 | None | 0 |
|  | 2 | None | 0 |
|  | 3 | None | 0 |
| NAS | 1 | None | 0 |
|  | 2 | None | 0 |
|  | 3 | None | 0 |
| Positive control  (natural rubber latex) | 1 | Severe | 4 |
|  | 2 | Severe | 4 |
|  | 3 | Severe | 4 |

- **Quantitative evaluation**

Viability greater than 70% was observed in the cultures treated with NAS, negative control. The positive control performed as expected (Table S2).

**Table S2 Quantitative evaluation (NR absorbance at 550 nm)**

| **Sample** | **Replicate 1** | **Replicate 2** | **Replicate 3** | **Mean** | **Viability (%)** | **Cytotoxicity**  **(%)** |
| --- | --- | --- | --- | --- | --- | --- |
| Negative control | 0.88 | 0.90 | 0.90 | 0.89 | 100.00 | 0.00 |
| NAS | 0.86 | 0.85 | 0.81 | 0.84 | 94.38 | 5.62 |
| Positive control | 0.14 | 0.10 | 0.11 | 0.12 | 13.48 | 86.52 |

1. **Skin sensitization using the guinea pig maximization test**

- **Mortality & morbidity**

No mortality or morbidity occurred in any of the animals used in this study.

Body weight A gradual increase in the body weight was observed in all the animals at the end of the experiment. Body weight of animals recorded prior to dosing and end of the experiment are given in Table S3.

**Table S3 Individual body weight of all animals in skin sensitization test**

| **Test/Group** | **Sex** | **Animal No.** | **Weight (in grams)** | | |
| --- | --- | --- | --- | --- | --- |
|  |  |  | **At the time of dosing** | **At the end of experiment** | **Increase in weight** |
| Preliminary  Test | M | 01 | 376.34 | 378.36 | 2.02 |
|  |  | 02 | 345.09 | 347.00 | 1.91 |
|  |  | 03 | 358.41 | 360.57 | 2.16 |
|  |  | 04 | 345.19 | 347.16 | 1.97 |
|  |  | Mean ± SD | 356.26 ± 14.78 | 358.27 ± 14.83 | 2.02 ± 0.11 |
| Main Test G1 Negative control  (NSS) | M | 01 | 341.25 | 373.76 | 32.51 |
|  |  | 02 | 368.37 | 400.29 | 31.92 |
|  |  | 03 | 376.13 | 409.15 | 33.02 |
|  |  | 04 | 348.29 | 379.99 | 31.70 |
|  |  | 05 | 365.39 | 394.17 | 28.78 |
|  |  | Mean ± SD | 359.89 ± 14.56 | 391.47 ± 14.52 | 31.59 ± 1.65 |
| Main Test G2 (NAS) | M | 06 | 346.24 | 379.10 | 32.86 |
|  |  | 07 | 341.37 | 374.38 | 33.01 |
|  |  | 08 | 368.92 | 398.32 | 29.40 |
|  |  | 09 | 357.80 | 386.60 | 28.80 |
|  |  | 10 | 376.60 | 406.69 | 30.09 |
|  |  | 11 | 351.63 | 383.41 | 31.78 |
|  |  | 12 | 348.87 | 380.45 | 31.58 |
|  |  | 13 | 361.42 | 393.71 | 32.29 |
|  |  | 14 | 339.02 | 368.13 | 29.11 |
|  |  | 15 | 372.27 | 402.35 | 30.08 |
|  |  | Mean ± SD | 356.41 ± 13.14 | 387.31 ± 12.60 | 30.90 ± 1.59 |

M - Male; SD- Standard Deviation

- **Grading of skin reactions**

Grading of skin reactions performed at 24 h and 48 h after removing the challenge patch are given in Table S4. No sensitization reactions were observed in animals treated with the negative control (NSS). No evidence of sensitization was seen in any of the NAS treated animals, as no skin reactions were observed.

**Table S4 Grading of skin reaction**

| **Group** | **Sex** | **Animal No.** | **After 24 h exposure** | | | |
| --- | --- | --- | --- | --- | --- | --- |
| Preliminary  Test | M | 01 | 0 | | | |
|  |  | 02 | 0 | | | |
|  |  | 03 | 0 | | | |
|  |  | 04 | 0 | | | |
| **Group** | **Sex** | **Animal No.** | **Magnusson and Kligman Scale** | | | |
|  |  |  | **24 h** | | **48 h** | |
|  |  |  | **C** | **T** | **C** | **T** |
| Negative control  (NSS) | M | 01 | 0 | 0 | 0 | 0 |
|  |  | 02 | 0 | 0 | 0 | 0 |
|  |  | 03 | 0 | 0 | 0 | 0 |
|  |  | 04 | 0 | 0 | 0 | 0 |
|  |  | 05 | 0 | 0 | 0 | 0 |
| NAS | M | 06 | 0 | 0 | 0 | 0 |
|  |  | 07 | 0 | 0 | 0 | 0 |
|  |  | 08 | 0 | 0 | 0 | 0 |
|  |  | 09 | 0 | 0 | 0 | 0 |
|  |  | 10 | 0 | 0 | 0 | 0 |
|  |  | 11 | 0 | 0 | 0 | 0 |
|  |  | 12 | 0 | 0 | 0 | 0 |
|  |  | 13 | 0 | 0 | 0 | 0 |
|  |  | 14 | 0 | 0 | 0 | 0 |
|  |  | 15 | 0 | 0 | 0 | 0 |

M-Male; C- Control site; T- Treated site; h- hour

1. **Intracutaneous reactivity potentials in New Zealand white rabbits**

- **Mortality & Morbidity**

No mortality or morbidity occurred in any of the animals throughout the experiment.

- **Body Weight**

A gradual increase in body weight was observed in all the animals at the end of experiment. Individual body weight of the animals is given in Table S5.

**Table S5 Individual body weights of New Zealand white rabbits**

| **Animal No.** | **Sex** | **Body weight (g)** | |
| --- | --- | --- | --- |
|  |  | **Initial** | **Final** |
| 1 | M | 2204.0 | 2238.1 |
| 2 |  | 2390.4 | 2427.9 |
| 3 |  | 2162.1 | 2190.6 |

M-Male

- **Clinical Observation**

No signs of ill health or overt toxicity were observed in any of the test animals.

- **Scoring of Skin Reaction**

NAS injected sites showed erythema at 24 h, 48 h and 72 h and no reactions were observed at the control sites. Grading of skin reactions for individual animals were given in Table S6. The difference of the mean skin reaction scores for the NAS and control was 0.4 (Table S7). The overall difference between the mean reaction grades (erythema) for the NAS and the control is less than 1.0.

**Table S6 Grading of skin reactions for individual New Zealand White rabbits**

| **Time points** | | | **24 h** | | | | | | **48 h** | | | | | | **72 h** | | | | |
| --- | --- | --- | --- | --- | --- | --- | --- | --- | --- | --- | --- | --- | --- | --- | --- | --- | --- | --- | --- |
| **Animal  No.** | **Sex** | **Sites** | **NAS** | |  | **Negative Control** | |  | **NAS** | |  | **Negative Control** | |  | **NAS** | |  | **Negative Control** | |
|  |  |  | **E** | **O** |  | **E** | **O** |  | **E** | **O** |  | **E** | **O** |  | **E** | **O** |  | **E** | **O** |
| **1** | **Male** | 1  2  3  4  5 | 1  0  0  1  1 | 0  0  0  0  0 |  | 0  0  0  0  0 | 0  0  0  0  0 |  | 1  1  0  0  1 | 0  0  0  0  0 |  | 0  0  0  0  0 | 0  0  0  0  0 |  | 0  0  0  0  0 | 0  0  0  0  0 |  | 0  0  0  0  0 | 0  0  0  0  0 |
|  |  | E+O | 3 | |  | 0 | |  | 3 | |  | 0 | |  | 0 | |  | 0 | |
|  |  | E+O/  5 sites | 0.6 | |  | 0 | |  | 0.6 | |  | 0 | |  | 0 | |  | 0 | |
| **2** |  | 1  2  3  4  5 | 0  1  1  0  0 | 0  0  0  0  0 |  | 0  0  0  0  0 | 0  0  0  0  0 |  | 1  1  0  0  0 | 0  0  0  0  0 |  | 0  0  0  0  0 | 0  0  0  0  0 |  | 0  0  0  1  1 | 0  0  0  0  0 |  | 0  0  0  0  0 | 0  0  0  0  0 |
|  |  | E+O | 2 | |  | 0 | |  | 2 | |  | 0 | |  | 2 | |  | 0 | |
|  |  | E+O/ 5 sites | 0.4 | |  | 0 | |  | 0.4 | |  | 0 | |  | 0.4 | |  | 0 | |
| **3** |  | 1  2  3  4  5 | 1  1  0  0  1 | 0  0  0  0  0 |  | 0  0  0  0  0 | 0  0  0  0  0 |  | 0  0  0  1  0 | 0  0  0  0  0 |  | 0  0  0  0  0 | 0  0  0  0  0 |  | 0  0  0  0  0 | 0  0  0  0  0 |  | 0  0  0  0  0 | 0  0  0  0  0 |
|  |  | E+O | 3 | |  | 0 | |  | 1 | |  | 0 | |  | 0 | |  | 0 | |
|  |  | E+O/  5 sites | 0.6 | |  | 0 | |  | 0.2 | |  | 0 | |  | 0 | |  | 0 | |

E, Erythema; O, Oedema

**Table S7 Calculation of skin reactions - Overall mean score and difference**

| **Negative control** | **NAS** | **Negative control** | **Overall difference** |
| --- | --- | --- | --- |
|  | **E+O** | **E+O** | **NAS - negative control** |
| Physiological saline | 0.4 | 0 | 0.4 |

E, Erythema; O, Oedema

1. **Acute systemic toxicity study via intranasal in mice**

- **Mortality & Morbidity**

No mortality or morbidity were observed in any of the animals used in this study.

- **Body Weight**

A gradual increase in body weight was observed in all animals at the end of experiment. Individual body weight of the animals is given in Table S8.

**Table S8 Individual body weights**

| **Group No.** | **Animal No.** | **Sex** | **Weight (in grams)** | | | |
| --- | --- | --- | --- | --- | --- | --- |
|  |  |  | **Days** | | | |
|  |  |  | **Day 0** | **24 h** | **48 h** | **72 h** |
| Negative control  (NSS) | 1 | M | 17.88 | 18.08 | 18.24 | 18.40 |
|  | 2 |  | 18.45 | 18.57 | 18.77 | 18.94 |
|  | 3 |  | 18.76 | 18.88 | 18.99 | 19.19 |
|  | 4 |  | 20.57 | 20.75 | 20.89 | 21.06 |
|  | 5 |  | 21.26 | 21.43 | 21.61 | 21.72 |
| NAS | 6 | M | 18.07 | 18.19 | 18.33 | 18.43 |
|  | 7 |  | 18.66 | 18.79 | 18.94 | 19.13 |
|  | 8 |  | 19.40 | 19.54 | 19.70 | 19.87 |
|  | 9 |  | 20.95 | 21.07 | 21.23 | 21.36 |
|  | 10 |  | 21.29 | 21.47 | 21.57 | 21.67 |

M - Male

- **Clinical Observation**

No signs of ill health or overt toxicity were observed in any of the animals (Table S9).

**Table S9 Clinical Observations**

| **Group No.** | **Animal No.** | **Sex** | **Observation at** | | | | | |
| --- | --- | --- | --- | --- | --- | --- | --- | --- |
|  |  |  | **0 h** | **30 min** | **4 h** | **24 h** | **48 h** | **72 h** |
| Negative control  (NSS) | 1 | M | N | N | N | N | N | N |
|  | 2 |  | N | N | N | N | N | N |
|  | 3 |  | N | N | N | N | N | N |
|  | 4 |  | N | N | N | N | N | N |
|  | 5 |  | N | N | N | N | N | N |
| NAS | 6 | M | N | N | N | N | N | N |
|  | 7 |  | N | N | N | N | N | N |
|  | 8 |  | N | N | N | N | N | N |
|  | 9 |  | N | N | N | N | N | N |
|  | 10 |  | N | N | N | N | N | N |

M - Male; h-hour; min- minutes; N-Normal

- **Gross Pathology, Clinical Pathology, and Histopathology**

Since no abnormal clinical signs were observed, no gross pathology, clinical pathology or histopathology were conducted.

1. **28-day subacute systemic toxicity study via oral administration in rats**

- **Mortality & Morbidity**

No morbidity or mortality occurred in any of the animals in both the control and treated group throughout the study.

- **Body Weight**

A gradual increase in body weight was observed in all the animals and no significant changes were observed between the treated and control groups as shown in Table S10.

**Table S10 Summary of weekly body weight (g)**

| **Group** | **Days** | | | | |
| --- | --- | --- | --- | --- | --- |
|  | **0** | **7** | **14** | **21** | **28** |
| Negative control  Male  N = 06 | 186.07 | 205.73 | 225.08 | 244.25 | 263.61 |
|  | ± | ± | ± | ± | ± |
|  | 8.502 | 9.330 | 8.465 | 9.273 | 8.898 |
| Negative control  Female  N = 06 | 181.36 | 202.14 | 222.34 | 242.06 | 262.41 |
|  | ± | ± | ± | ± | ± |
|  | 6.732 | 8.191 | 7.872 | 8.344 | 9.658 |
| NAS  Male  N = 06 | 187.36 | 207.94 | 228.65 | 250.61 | 270.99 |
|  | ± | ± | ± | ± | ± |
|  | 7.350 | 6.504 | 5.167 | 6.977 | 6.138 |
| NAS  Female  N = 06 | 182.38 | 201.12 | 220.21 | 238.98 | 257.12 |
|  | ± | ± | ± | ± | ± |
|  | 6.877 | 7.429 | 7.577 | 9.434 | 9.863 |

Values are expressed as Mean ± SD; N, Number of animals.

- **Feed consumption**

None of the animals in treated group showed significant difference in the feed consumption when compared to the control group as shown in Table S11.

**Table S11 Summary of weekly feed consumption (g)**

| **Group/Sex** | **Days** | | | |
| --- | --- | --- | --- | --- |
|  | **7** | **14** | **21** | **28** |
| Negative control  Male  N = 06 | 94.70 | 99.45 | 97.86 | 98.77 |
|  | ± | ± | ± | ± |
|  | 3.737 | 6.117 | 3.388 | 4.728 |
| Negative control  Female  N = 06 | 96.73 | 98.90 | 100.51 | 98.04 |
|  | ± | ± | ± | ± |
|  | 3.096 | 5.077 | 4.144 | 3.906 |
| NAS  Male  N = 06 | 95.95 | 99.26 | 95.36 | 98.39 |
|  | ± | ± | ± | ± |
|  | 5.377 | 3.163 | 3.615 | 5.488 |
| NAS  Female  N = 06 | 98.77 | 97.38 | 99.19 | 97.75 |
|  | ± | ± | ± | ± |
|  | 4.728 | 4.902 | 5.394 | 6.796 |

Values are expressed as Mean ± SD

- **Clinical Observation**

None of the animals from any group showed any clinical signs of toxicity during the course of the study.

- **Urinalysis**

No abnormal difference was observed in the urine analysis performed in the samples and all the parameters were within acceptable range when compared to the control animals see in Table S12.

- **Hematology and Clinical Biochemistry**

In male animals, no significant changes were observed in haematology, biochemical parameters and electrolytes when compared with the control group.

In female animals, no significant changes were observed in haematology (except WBC values), biochemical parameters and electrolytes. A significant difference was observed in the WBC values (p=0.0027) between the control and treated groups. Since the values were within the normal physiological range, these changes were considered not related to the NAS as shown in Table S13.

- **Gross Pathology**

No NAS and control item related gross pathological changes were observed. Most tissues were macroscopically unremarkable, and the other findings seen were generally consistent with the usual pattern of findings in animals of this strain and age as shown in Table S14.

- **Organ Weights**

In male animals, no significant changes were observed in absolute and relative organ weights (except relative organ weights of adrenal and epididymis) when compared with the control group. A significant difference was observed in the relative organ weight of adrenal (p=0.0269) and epididymis (p=0.0277) between the control and treated groups. Since the values were within the normal physiological range, these changes were considered not related to the NAS as shown in Table S15.

- **Histopathology**

No NAS and control item related histopathological changes were observed. Other observed microscopic findings were generally infrequent, of a minor nature and consistent with the usual pattern of findings in animals of this strain and age. All of the observed changes were considered to be spontaneous background lesions and not related to adverse effects of control or NAS as shown in Table S16.

Since no abnormal findings were observed in Tier I organs/tissues and in clinical pathology analysis (clinical chemistry and haematology), the histopathology analysis of Tier II organs/tissues were not performed.

**Table S12 Summary of Urinalysis**

| **ORGANS** | **Parameter** | **GROUPS** | | | |
| --- | --- | --- | --- | --- | --- |
|  |  | **Negative control** | **Negative control** | **NAS** | **NAS** |
|  |  | **Male** | **Female** | **Male** | **Female** |
|  |  | **6 animals** | **6 animals** | **6 animals** | **6 animals** |
| Volume | Mean | 6.97 | 6.65 | 6.28 | 7.30 |
|  | SD | 0.871 | 0.536 | 0.546 | 1.126 |
| Colour | Pale Yellow | 3 | 2 | 3 | 3 |
|  | Yellow | 3 | 3 | 2 | 2 |
|  | Brown | 0 | 1 | 1 | 1 |
| Urobilinogen | 3.3 | 4 | 4 | 3 | 3 |
|  | 16 | 2 | 1 | 2 | 1 |
|  | 33 | 0 | 1 | 1 | 2 |
| Bilirubin | Positive | 0 | 0 | 0 | 0 |
|  | Negative | 6 | 6 | 6 | 6 |
| Ketone | Negative | 6 | 6 | 6 | 6 |
|  | 0.5 | 0 | 0 | 0 | 0 |
| Blood cells | Negative | 6 | 6 | 6 | 6 |
| Protein | Negative | 1 | 1 | 1 | 1 |
|  | 0.2 | 2 | 4 | 4 | 3 |
|  | 0.3 | 3 | 1 | 1 | 2 |
| Nitrite | Negative | 6 | 6 | 6 | 6 |
| Leucocytes | Negative | 6 | 6 | 6 | 6 |
| Glucose | Negative | 6 | 6 | 6 | 6 |
| Gravity | 1.010 | 2 | 1 | 2 | 2 |
|  | 1.015 | 0 | 3 | 2 | 1 |
|  | 1.020 | 3 | 1 | 1 | 1 |
|  | 1.025 | 1 | 1 | 1 | 2 |
|  | ≥1.030 | 0 | 0 | 0 | 0 |
| pH | 6.0 | 1 | 0 | 1 | 0 |
|  | 6.5 | 3 | 2 | 2 | 1 |
|  | 7.0 | 2 | 2 | 2 | 2 |
|  | 7.5 | 0 | 1 | 1 | 1 |
|  | 8.0 | 0 | 1 | 0 | 1 |
|  | 8.5 | 0 | 0 | 0 | 1 |
| Microscopic Examination |  | NAD | NAD | NAD | NAD |

NAD – No Abnormality Detected

**Table S13 Summary of hematology measurements**

| **Group** | **RBC**  **(10^6^/µL)** | **Hb**  **(g/dL)** | **HCT**  **(%)** | **WBC**  **(10^3^/µL)** | **PLT**  **(10^3^/µL)** | **Differential count** | | | **MCH**  **(pg)** | **MCHC**  **(g/dL)** | **MCV**  **(fL)** |
| --- | --- | --- | --- | --- | --- | --- | --- | --- | --- | --- | --- |
|  |  |  |  |  |  | **L** | **M** | **G** |  |  |  |
|  |  |  |  |  |  | **(%)** | **(%)** | **(%)** |  |  |  |
| Negative control  Male  N = 06 | 8.08 | 13.95 | 41.15 | 12.42 | 676.17 | 78.05 | 3.92 | 18.03 | 17.32 | 33.97 | 50.97 |
|  | ± | ± | ± | ± | ± | ± | ± | ± | ± | ± | ± |
|  | 0.423 | 0.742 | 2.015 | 1.359 | 90.19 | 7.302 | 0.240 | 7.278 | 1.338 | 2.574 | 2.023 |
| Negative control Female  N = 06 | 7.63 | 13.62 | 42.17 | 13.63 | 646.50 | 72.27 | 2.80 | 24.93 | 17.90 | 32.33 | 55.40 |
|  | ± | ± | ± | ± | ± | ± | ± | ± | ± | ± | ± |
|  | 0.467 | 0.741 | 1.468 | 1.063 | 65.06 | 6.178 | 0.684 | 6.550 | 1.393 | 2.023 | 3.125 |
| NAS  Male  N = 06 | 7.69 | 14.25 | 39.50 | 12.35 | 632.33 | 76.47 | 3.32 | 20.22 | 18.68 | 36.30 | 51.62 |
|  | ± | ± | ± | ± | ± | ± | ± | ± | ± | ± | ± |
|  | 0.699 | 0.946 | 3.010 | 1.677 | 74.93 | 4.219 | 0.898 | 4.770 | 2.319 | 4.077 | 4.573 |
| NAS  Female  N = 06 | 7.39 | 13.07 | 41.92 | 11.33 | 706.17 | 74.70 | 3.43 | 21.87 | 17.85 | 31.27 | 57.05 |
|  | ± | ± | ± | ± | ± | ± | ± | ± | ± | ± | ± |
|  | 0.648 | 1.236 | 2.949 | 0.94 | 43.64 | 5.519 | 1.042 | 6.353 | 2.935 | 3.204 | 6.344 |

Values are expressed as Mean ± SD; Hb, hemoglobin; RBC, red blood cell count; HCT, hematocrit; MCH, mean corpuscular haemoglobin; MCHC, mean corpuscular haemoglobin concentration; MCV, mean corpuscular volume; WBC, white blood cell count; L, lymphocyte percentage; M, monocyte percentage; G, granulocyte percentage; PLT, platelet count; CT, clotting time.

**Table S14 Summary of gross pathology**

| **ORGANS** | **LESIONS** | **Groups** | | | |
| --- | --- | --- | --- | --- | --- |
|  |  | **Negative control** | **Negative control** | **NAS** | **NAS** |
|  |  | **Male** | **Female** | **Male** | **Female** |
|  |  | **6 animals** | **6 animals** | **6 animals** | **6 animals** |
| **Adrenals** | NAD | - | - | - | - |
| **Aorta** | NAD | - | - | - | - |
| **Bone marrow (Sternum)** | NAD | - | - | - | - |
| **Brain** | NAD | - | - | - | - |
| **Caecum** | NAD | - | - | - | - |
| **Colon** | NAD | - | - | - | - |
| **Duodenum** | NAD | - | - | - | - |
| **Epididymis** | NAD | - | NA | - | NA |
| **Oesophagus** | NAD | - | - | - | - |
| **Eyes** | NAD | - | - | - | - |
| **Femur** | NAD | - | - | - | - |
| **Heart** | NAD | - | - | - | - |
| **Ileum** | NAD | - | - | - | - |
| **Jejunum** | NAD | - | - | - | - |
| **Kidneys** | Irregular areas of red discoloration – diffuse | - | - | 1 | - |
| **Liver** | Irregular areas of red discoloration – diffuse | - | 1 | - | 1 |
| **Lungs and Bronchi** | Irregular areas of red discoloration – diffuse | 1 | 1 | 1 | - |
| **Lymph nodes** | NAD | - | - | - | - |
| **Mammary gland** | NAD | NA | - | NA | - |
| **Skeletal muscle** | NAD | - | - | - | - |
| **Sciatic nerve** | NAD | - | - | - | - |
| **Ovaries** | NAD | NA | - | NA | - |
| **Pancreas** | NAD | - | - | - | - |
| **Parathyroid** | NAD | - | - | - | - |
| **Pituitary** | NAD | - | - | - | - |
| **Prostate** | NAD | - | NA | - | NA |
| **Rectum** | NAD | - | - | - | - |
| **Salivary glands** | NAD | - | - | - | - |
| **Seminal vesicles** | NAD | - | NA | - | NA |
| **Skin** | NAD | - | - | - | - |
| **Spinal cord** | NAD | - | - | - | - |
| **Femur** | NAD | - | - | - | - |
| **Spleen** | NAD | - | - | - | - |
| **Stomach** | NAD | - | - | - | - |
| **Testes** | NAD | - | NA | - | NA |
| **Thymus** | NAD | - | - | - | - |
| **Thyroid** | NAD | - | - | - | - |
| **Trachea** | NAD | - | - | - | - |
| **Urinary bladder** | NAD | - | - | - | - |
| **Uterus** | NAD | NA | - | NA | - |
| **Vagina** | NAD | NA | - | NA | - |

NAD – No Abnormality Detected, NA – Not Applicable.

**Table S15 Summary of relative organ weights (%)**

| **Group** | **Adrenal** | **Thymus** | **Spleen** | **Heart** | **Brain** | **Kidney** | **Liver** | **Testes* / Ovary#** | **Epididymis* / Uterus#** |
| --- | --- | --- | --- | --- | --- | --- | --- | --- | --- |
| **Negative control**  **Male**  **N = 06** | 0.03  ±  0.004 | 0.13  ±  0.032 | 0.29  ±  0.070 | 0.38  ±  0.119 | 0.68  ±  0.044 | 0.64  ±  0.061 | 3.79  ±  0.464 | 0.49  ±  0.107 | 0.39  ±  0.016 |
| **Negative control**  **Female**  **N = 06** | 0.02  ±  0.005 | 0.13  ±  0.017 | 0.34  ±  0.065 | 0.33  ±  0.063 | 0.68  ±  0.068 | 0.66  ±  0.067 | 3.66  ±  0.538 | 0.059  ±  0.012 | 0.31  ±  0.036 |
| **NAS**  **Male**  **N = 06** | 0.02  ±  0.004 | 0.11  ±  0.039 | 0.35  ±  0.093 | 0.35  ±  0.118 | 0.64  ±  0.036 | 0.64  ±  0.020 | 3.63  ±  0.682 | 0.50  ±  0.118 | 0.37  ±  0.018 |
| **NAS**  **Female**  **N = 06** | 0.02  ±  0.005 | 0.12  ±  0.029 | 0.34  ±  0.076 | 0.35  ±  0.079 | 0.70  ±  0.049 | 0.72  ±  0.058 | 3.56  ±  0.223 | 0.06  ±  0.005 | 0.32  ±  0.073 |

Values are expressed in grams as Mean ± SD; * for males; # for females.

**Table S16 Summary of** **histopathology**

| **ORGANS** | **LESIONS** | **GROUPS** | | | |
| --- | --- | --- | --- | --- | --- |
|  |  | **Negative control** | **Negative control** | **NAS** | **NAS** |
|  |  | **Male** | **Female** | **Male** | **Female** |
|  |  | **6 animals** | **6 animals** | **6 animals** | **6 animals** |
| **Heart** | NSML | - | - | - | - |
| **Liver** | Infiltrate, inflammatory cells, focal, minimal | 1 | 1 | - | - |
|  | Pigments, brown to black, diffuse, minimal | - | - | 1 | - |
|  | Congestion, sinusoidal, diffuse, minimal | 1 | 2 | 1 | 1 |
| **Adrenals** | Congestion, diffuse, minimal | 1 | - | 0 | - |
|  | Vacuolation, cytoplasmic, diffuse, cortex, Zona fasciculata, mild | - | - | - | 1 |
| **Kidneys** | Congestion, diffuse, minimal | - | 1 | 1 | - |
|  | Infiltrate, mononuclear cells, interstitial, multifocal, cortex, mild | 1 | - | 1 | 1 |
| **Skin** | NSML | - | - | - | - |
| **Spleen** | Pigments, brown to black, multifocal, red pulp, minimal | - | 1 | 1 | 1 |
| **Skeletal muscle** | NSML | - | - | - | - |
| **Brain** | NSML | - | - | - | - |
| **Testes** | NSML | - | NA | - | NA |
| **Ovaries** | NSML | NA | - | NA | - |
| **Lungs and Bronchi** | Infiltrate, mononuclear cells, perivascular, multifocal, minimal | - | 1 | 1 | 0 |
|  | Congestion, diffuse, minimal | 1 | 1 | 2 | - |
|  | Congestion and oedema, diffuse, minimal | - | 1 | 1 | - |
|  | Infiltrate, mononuclear cells, perivascular, multifocal, mild  Congestion and oedema, diffuse, minimal | 1 | - | 1 | 1 |
| **Femur** | NSML | - | - | - | - |
| **Bone marrow (sternum)** | NSML | - | - | - | - |

NSML – No significant microscopic lesions; NA – Not applicable.
