# Supplementary material for: Assessment of safety and intranasal neutralizing antibodies of HPMC-based human anti-SARS-CoV-2 IgG1 nasal spray in healthy volunteers
Source: Sci Rep. 2023 Sep 20;13:15648. doi: 10.1038/s41598-023-42539-7 (PMC10511465; doi:10.1038/s41598-023-42539-7)
Supplement: Supplementary file 2 — Supplementary Information 2. [file 41598_2023_42539_MOESM2_ESM.docx]

**Supplemental Data S2**

Quantification of human IgG1 anti-SARS-CoV-2 antibodies in rat serum after intranasal application of NAS.


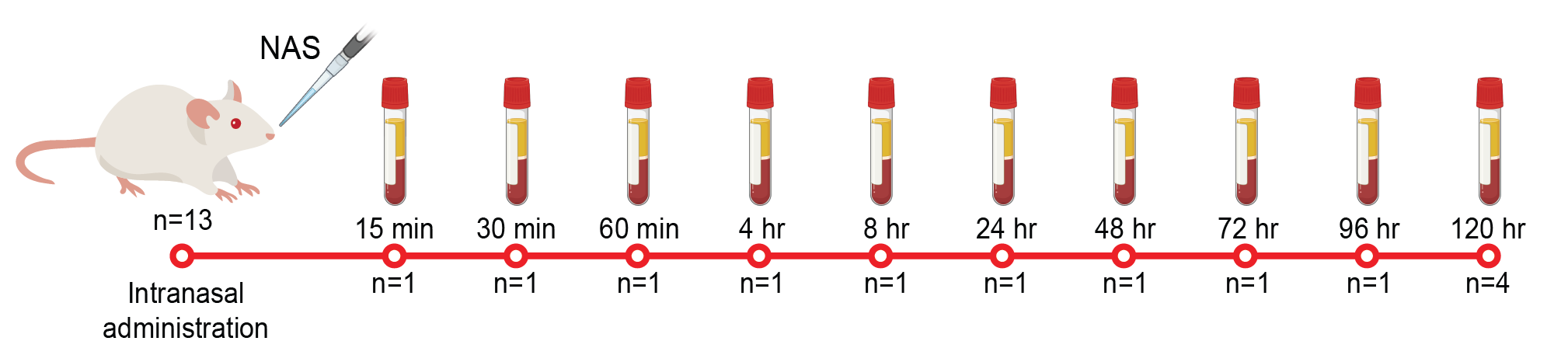


Figure S1 Illustration demonstrates the blood collection schedule in rats after NAS application.


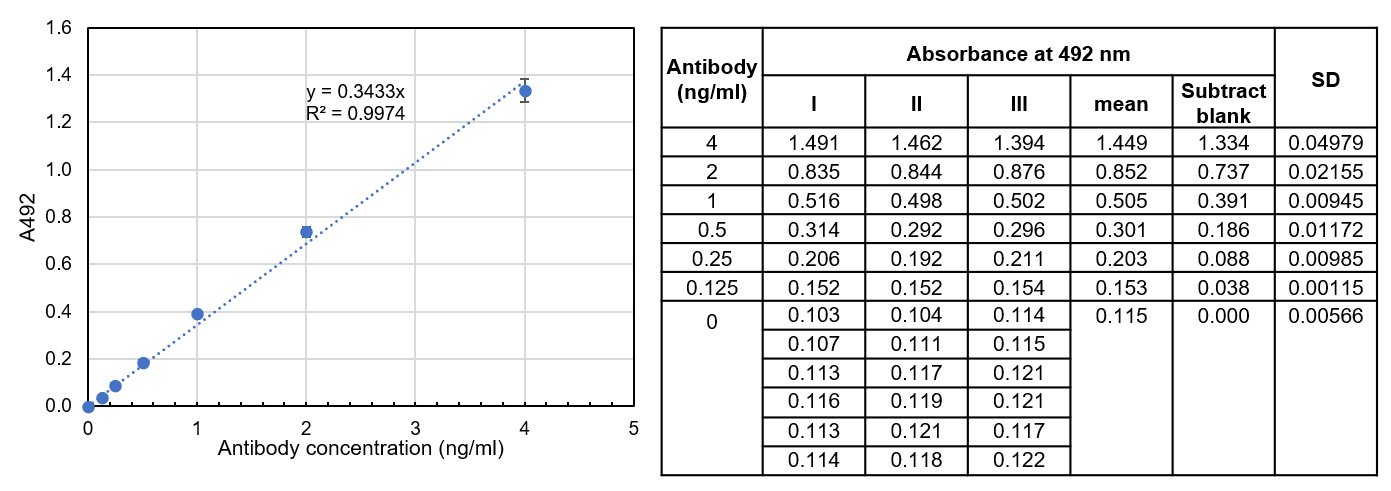


Figure S2 A standard curve from ELISA data of rat serum that has been spiked with human IgG1 anti-SARS-CoV-2 antibodies.

**According to the standard curve above, LOQ calculation formula is:**

LOQ = A492 LOQ/0.3433

**Where** A492 LOQ = (A492 at 0 ng/ml) + 10*(SD of A492 at 0 ng/ml)

= 0.000 + (10*0.00566)

= 0.0566

**Thus** LOQ = 0.0566/0.3433

= 0.165 ng/ml

**Table S17 Quantification of human IgG1 anti-SARS-CoV-2 antibodies in rat serum**

| Time point | A492 | | | Mean A_492_ | A_492_  SD | A_492_ Subtract blank | Calculated  IgG1 level  (ng/ml) | Interpretation |
| --- | --- | --- | --- | --- | --- | --- | --- | --- |
|  | I | II | III |  |  |  |  |  |
| 15min | 0.137 | 0.116 | 0.111 | 0.121 | 0.01380 | 0.007 | 0.019 | <LOQ |
| 30min | 0.127 | 0.111 | 0.107 | 0.115 | 0.01058 | 0.000 | 0.001 | <LOQ |
| 60min | 0.129 | 0.117 | 0.116 | 0.121 | 0.00723 | 0.006 | 0.017 | <LOQ |
| 4hr | 0.120 | 0.116 | 0.111 | 0.116 | 0.00451 | 0.001 | 0.003 | <LOQ |
| 8hr | 0.117 | 0.104 | 0.100 | 0.107 | 0.00889 | -0.008 | -0.023 | <LOQ |
| 24hr | 0.127 | 0.113 | 0.110 | 0.117 | 0.00907 | 0.002 | 0.006 | <LOQ |
| 48hr | 0.151 | 0.151 | 0.154 | 0.152 | 0.00203 | 0.037 | 0.108 | <LOQ |
| 72hr | 0.146 | 0.136 | 0.135 | 0.139 | 0.00608 | 0.024 | 0.071 | <LOQ |
| 96hr | 0.135 | 0.121 | 0.118 | 0.125 | 0.00907 | 0.010 | 0.029 | <LOQ |
| 120hr  (n=4) | 0.144 | 0.129 | 0.125 | 0.133 | 0.01002 | 0.018 | 0.052 | <LOQ |
|  | 0.114 | 0.117 | 0.124 | 0.118 | 0.00513 | 0.004 | 0.010 | <LOQ |
|  | 0.101 | 0.104 | 0.109 | 0.105 | 0.00404 | -0.010 | -0.029 | <LOQ |
|  | 0.114 | 0.117 | 0.123 | 0.118 | 0.00458 | 0.003 | 0.009 | <LOQ |
