# Supplementary material for: Assessment of safety and intranasal neutralizing antibodies of HPMC-based human anti-SARS-CoV-2 IgG1 nasal spray in healthy volunteers
Source: Sci Rep. 2023 Sep 20;13:15648. doi: 10.1038/s41598-023-42539-7 (PMC10511465; doi:10.1038/s41598-023-42539-7)
Supplement: Supplementary file 3 — Supplementary Information 3. [file 41598_2023_42539_MOESM3_ESM.docx]

**Supplemental Data S3**

**Nasal sinuscopy images of participants in the NAS and placebo groups on days 0, 7, and 14**

**Nasal sinuscopy images are displayed in random order of participants.**

**Table S18 Nasal sinuscopy images from NAS group (n = 27)**

|  | Nostril | |  | Nostril | |
| --- | --- | --- | --- | --- | --- |
| C1 | Right | Left | C2 | Right | Left |
| Day 0 | 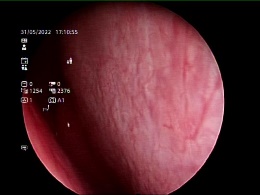 | 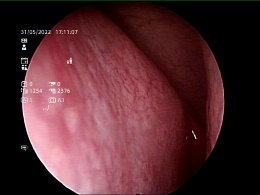 | Day 0 | 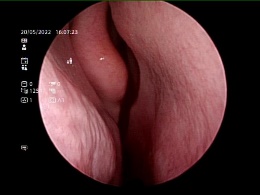 | 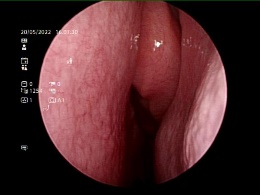 |
| Day 7 | 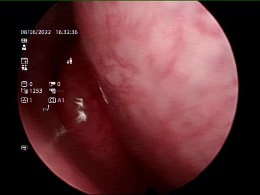 | 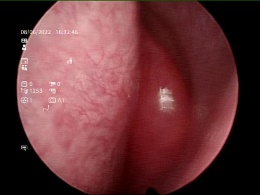 | Day 7 | 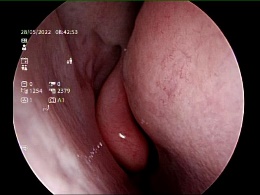 | 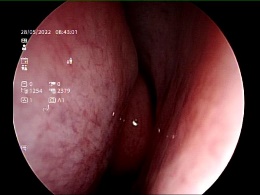 |
| Day 14 | 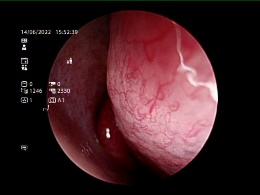 | 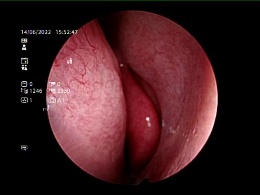 | Day 14 | 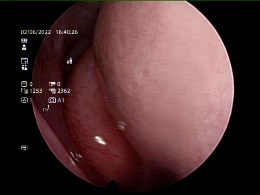 | 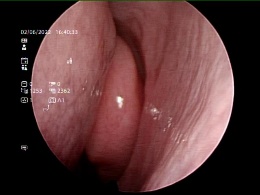 |
| C3 | Right | Left | C4 | Right | Left |
| Day 0 | 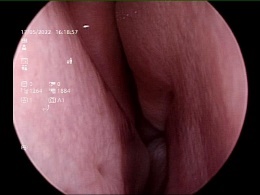 | 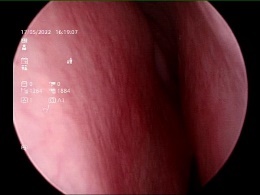 | Day 0 | 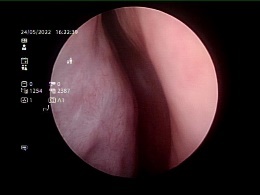 | 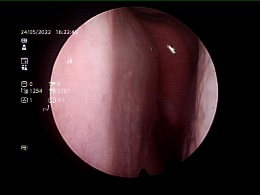 |
| Day 7 | 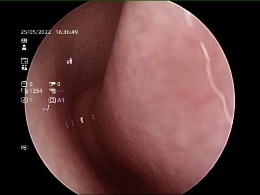 | 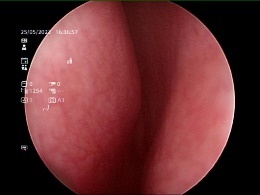 | Day 7 | 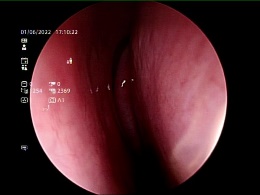 | 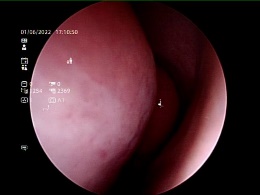 |
| Day 14 | 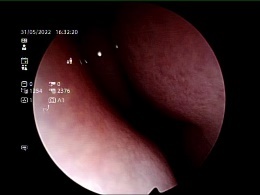 | 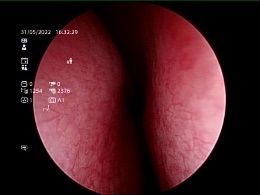 | Day 14 | 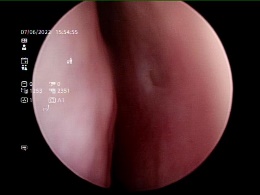 | 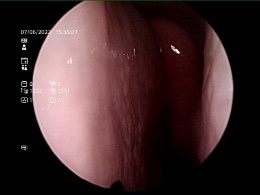 |
| C5 | Right | Left | C6 | Right | Left |
| Day 0 | 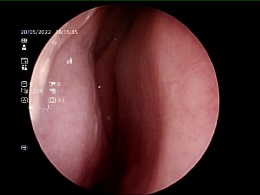 | 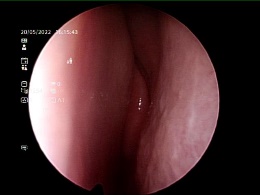 | Day 0 | 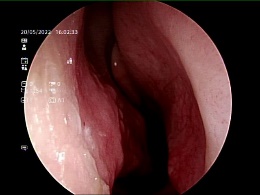 | 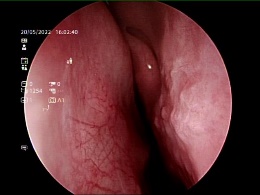 |
| Day 7 | 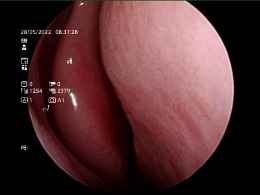 | 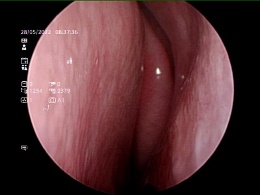 | Day 7 | 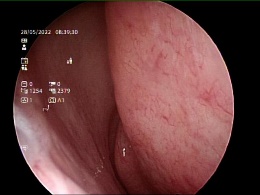 | 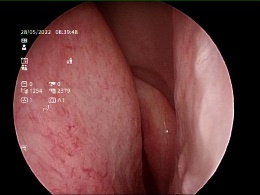 |
| Day 14 | 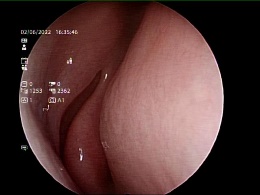 | 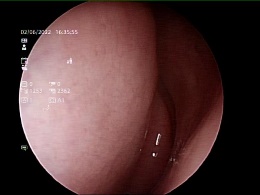 | Day 14 | 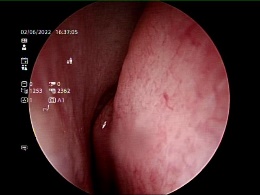 | 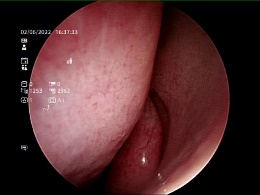 |

|  | Nostril | |  | Nostril | |
| --- | --- | --- | --- | --- | --- |
| C7 | Right | Left | C8 | Right | Left |
| Day 0 | 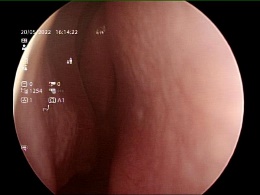 | 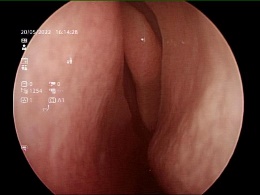 | Day 0 | 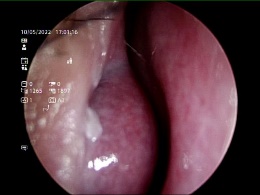 | 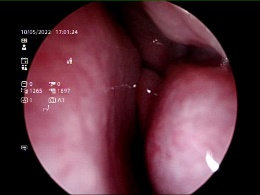 |
| Day 7 | 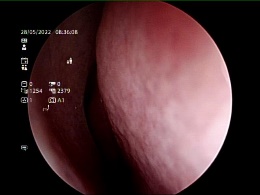 | 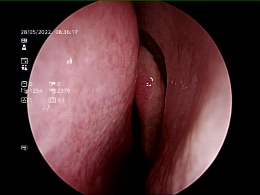 | Day 7 | 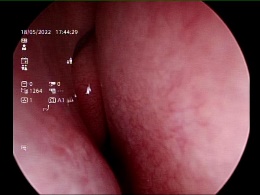 | 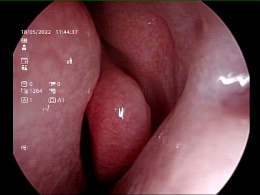 |
| Day 14 | 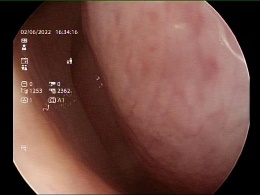 | 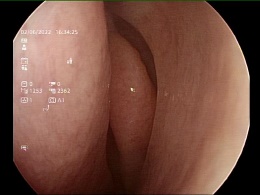 | Day 14 | 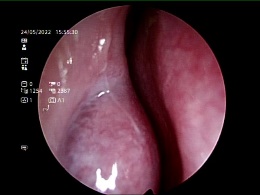 | 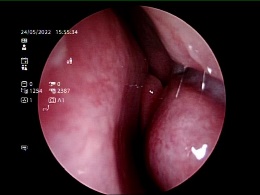 |
| C9 | Right | Left | C10 | Right | Left |
| Day 0 | 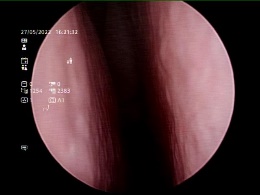 | 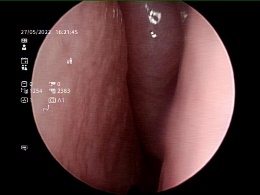 | Day 0 | 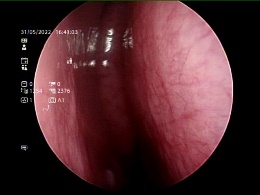 | 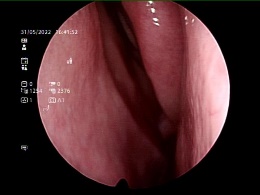 |
| Day 7 | 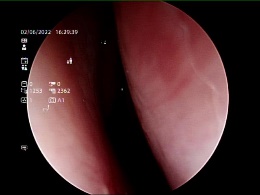 | 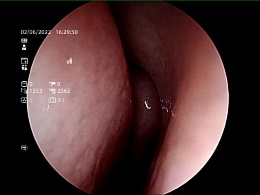 | Day 7 | 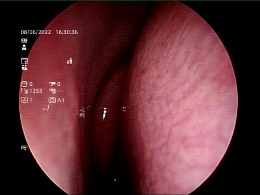 | 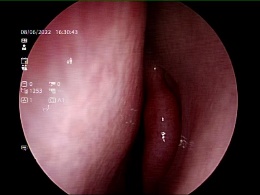 |
| Day 14 | 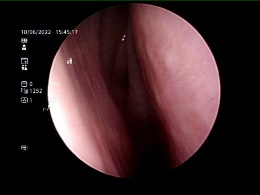 | 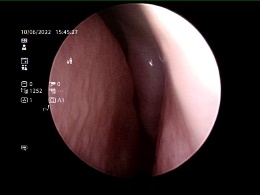 | Day 14 | 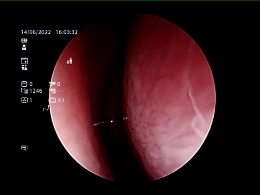 | 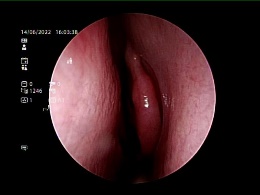 |
| C11 | Right | Left | C12 | Right | Left |
| Day 0 | 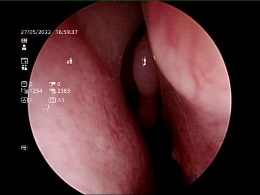 | 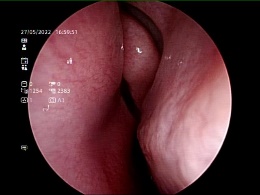 | Day 0 | 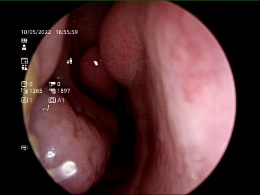 | 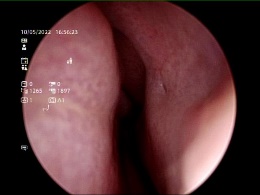 |
| Day 7 | 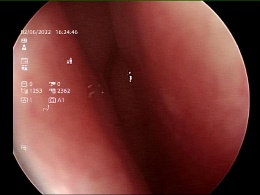 | 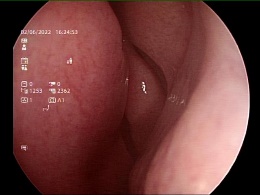 | Day 7 | 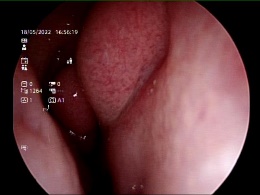 | 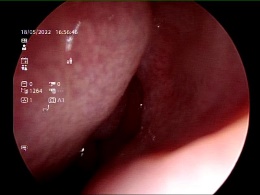 |
| Day 14 | 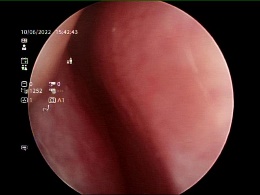 | 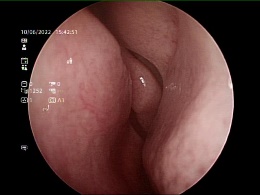 | Day 14 | 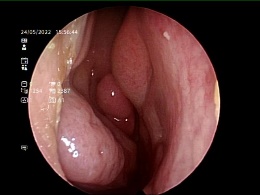 | 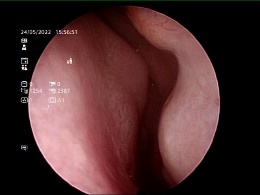 |

|  | Nostril | |  | Nostril | |
| --- | --- | --- | --- | --- | --- |
| C13 | Right | Left | C14 | Right | Left |
| Day 0 | 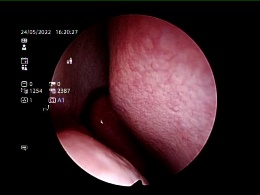 | 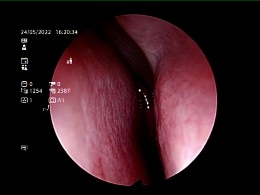 | Day 0 | 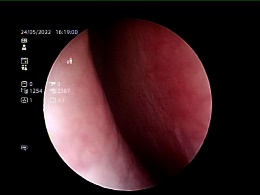 | 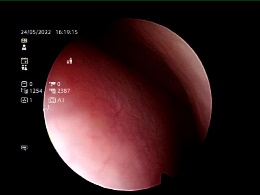 |
| Day 7 | 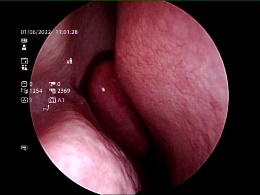 | 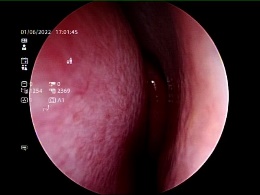 | Day 7 | 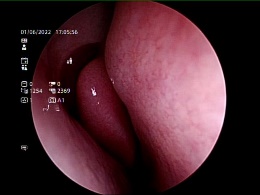 | 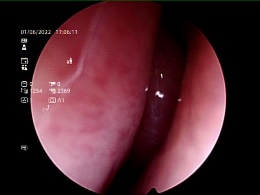 |
| Day 14 | 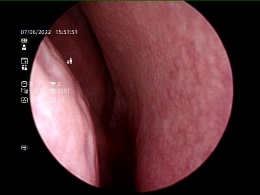 | 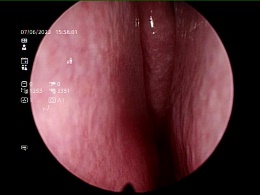 | Day 14 | 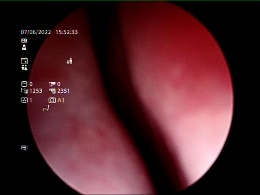 | 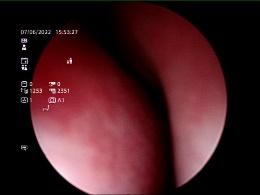 |
| C15 | Right | Left | C16 | Right | Left |
| Day 0 | 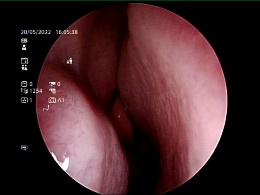 | 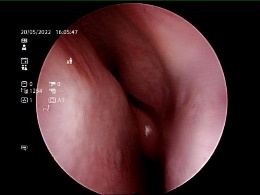 | Day 0 | 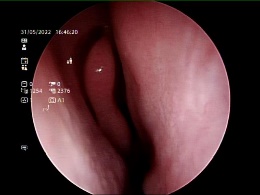 | 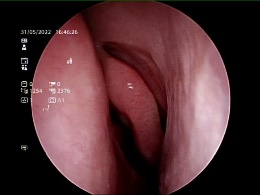 |
| Day 7 | 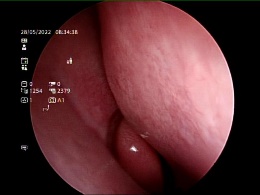 | 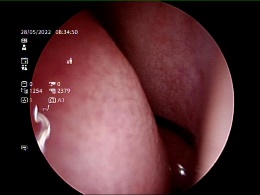 | Day 7 | 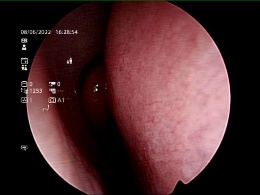 | 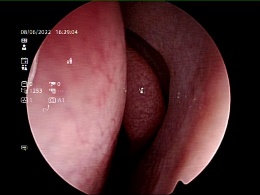 |
| Day 14 | 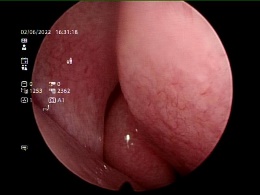 | 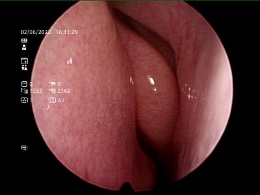 | Day 14 | 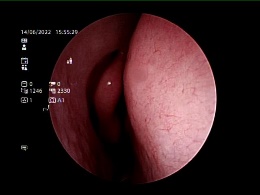 | 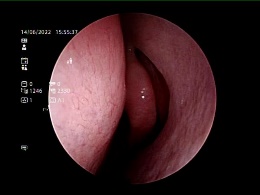 |
| C17 | Right | Left | C18 | Right | Left |
| Day 0 | 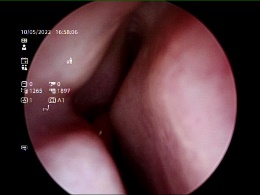 | 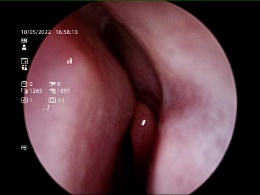 | Day 0 | 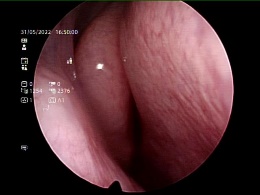 | 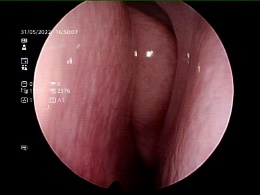 |
| Day 7 |  |  | Day 7 |  |  |
| Day 14 |  |  | Day 14 |  |  |

|  | Nostril | |  | Nostril | |
| --- | --- | --- | --- | --- | --- |
| C19 | Right | Left | C20 | Right | Left |
| Day 0 |  |  | Day 0 |  |  |
| Day 7 |  |  | Day 7 |  |  |
| Day 14 |  |  | Day 14 |  |  |
| C21 | Right | Left | C22 | Right | Left |
| Day 0 |  |  | Day 0 |  |  |
| Day 7 |  |  | Day 7 |  |  |
| Day 14 |  |  | Day 14 |  |  |
| C23 | Right | Left | C24 | Right | Left |
| Day 0 |  |  | Day 0 |  |  |
| Day 7 |  |  | Day 7 |  |  |
| Day 14 |  |  | Day 14 |  |  |

|  | Nostril | |  | Nostril | |
| --- | --- | --- | --- | --- | --- |
| C25 | Right | Left | C26 | Right | Left |
| Day 0 |  |  | Day 0 |  |  |
| Day 7 |  |  | Day 7 |  |  |
| Day 14 |  |  | Day 14 |  |  |
| C27 | Right | Left |  |  |  |
| Day 0 |  |  |  |  |  |
| Day 7 |  |  |  |  |  |
| Day 14 |  |  |  |  |  |

**Table S19 Nasal sinuscopy images from Placebo group (n = 9)**

|  | Nostril | |  | Nostril | |
| --- | --- | --- | --- | --- | --- |
| P1 | Right | Left | P2 | Right | Left |
| Day 0 |  |  | Day 0 |  |  |
| Day 7 |  |  | Day 7 |  |  |
| Day 14 |  |  | Day 14 |  |  |
| P3 | Right | Left | P4 | Right | Left |
| Day 0 |  |  | Day 0 |  |  |
| Day 7 |  |  | Day 7 |  |  |
| Day 14 |  |  | Day 14 |  |  |
| P5 | Right | Left | P6 | Right | Left |
| Day 0 |  |  | Day 0 |  |  |
| Day 7 |  |  | Day 7 |  |  |
| Day 14 |  |  | Day 14 |  |  |

|  | Nostril | |  | Nostril | |
| --- | --- | --- | --- | --- | --- |
| P7 | Right | Left | P8 | Right | Left |
| Day 0 |  |  | Day 0 |  |  |
| Day 7 |  |  | Day 7 |  |  |
| Day 14 |  |  | Day 14 |  |  |
| P9 | Right | Left |  |  |  |
| Day 0 |  |  |  |  |  |
| Day 7 |  |  |  |  |  |
| Day 14 |  |  |  |  |  |
