# Supplementary material for: Assessment of safety and intranasal neutralizing antibodies of HPMC-based human anti-SARS-CoV-2 IgG1 nasal spray in healthy volunteers
Source: Sci Rep. 2023 Sep 20;13:15648. doi: 10.1038/s41598-023-42539-7 (PMC10511465; doi:10.1038/s41598-023-42539-7)
Supplement: Supplementary file 4 — Supplementary Information 4. [file 41598_2023_42539_MOESM4_ESM.docx]

**Supplemental Data S4**

**Table S20 Detailed statistical report of the SARS-CoV-2 neutralizing antibodies in nasal fluids after nasal spray application in both the NAS and placebo groups.**

| **VOCs** | **Product** | **Timepoint of nasal fluid sample collection in relation to the study product application** | ***n*** | **Median of % inhibition**  **(IQR25%-IQR75%)** | **95% CI**  **of median** | **Wilcoxon test**  **(*P* value)** |
| --- | --- | --- | --- | --- | --- | --- |
| **Ancestral** | Placebo | Before | 4 | 7.69  (1.34 - 67.38) | 0 – 86.51 | 0.1875 |
|  |  | Immediately  after | 4 | 14.35  (3.25 - 75.39) | 0 – 95.27 |  |
|  | NAS | Before | 13 | 12.41  (3.56 - 27.39) | 0 – 33.63 | 1.2207E-4 |
|  |  | Immediately  after | 13 | 97.58  (97.11 - 97.67) | 96.93 – 97.72 |  |
|  | Placebo | Before | 5 | 24.16  (6.48 - 94.45) | 0 – 97.73 | 0.1563 |
|  |  | 6 hours after | 5 | 46.68  (33.58 - 86.97) | 20.72 – 97.86 |  |
|  | NAS | Before | 14 | 24.03  (12.2 - 34.9) | 4.06 – 38.36 | 6.1035E-05 |
|  |  | 6 hours after | 14 | 91.72  (76.2 - 97.04) | 66.43 – 97.29 |  |
| **Delta** | Placebo | Before | 4 | 2.98  (2.25 - 49.94) | 2.13 – 65.46 | 0.3125 |
|  |  | Immediately  after | 4 | 5.38  (0.53 - 65.7) | 0 – 84.73 |  |
|  | NAS | Before | 13 | 8.02  (1.35 - 30.07) | 0.83 – 35.06 | 1.2207E-4 |
|  |  | Immediately  after | 13 | 97.44  (96.88 - 97.57) | 96.61 – 97.62 |  |
|  | Placebo | Before | 5 | 35.01  (6.96 - 86.66) | 0 – 96.83 | 0.0938 |
|  |  | 6 hours after | 5 | 48.4  (23.64 - 86.17) | 23.08 – 97.63 |  |
|  | NAS | Before | 14 | 21.53  (3.979 - 23.23) | 0 – 24.04 | 6.1035E-05 |
|  |  | 6 hours after | 14 | 88.67  (73.01 - 96.31) | 70.83 – 96.92 |  |
| **Omicron BA.2** | Placebo | Before | 4 | 2.16  (0 - 33.58) | 0 – 43.33 | 0.1250 |
|  |  | Immediately  after | 4 | 12.5  (2.39 - 50.35) | 0 – 61.98 |  |
|  | NAS | Before | 13 | 3.64  (0 - 10.99) | 0 – 11.58 | 1.2207E-4 |
|  |  | Immediately  after | 13 | 94.65  (92.43 - 95.54) | 91.69 – 95.74 |  |
|  | Placebo | Before | 5 | 13.63  (3.14 - 50.38) | 0 – 70.69 | 0.0625 |
|  |  | 6 hours after | 5 | 17.77  (13.32 - 59.87) | 10.35 – 81.63 |  |
|  | NAS | Before | 14 | 8.59  (0.09 - 15.79) | 0.04 – 16.78 | 6.1035E-05 |
|  |  | 6 hours after | 14 | 70.6  (54.83 - 85.09) | 48.33 – 88.95 |  |
