# Supplementary material for: Assessment of safety and intranasal neutralizing antibodies of HPMC-based human anti-SARS-CoV-2 IgG1 nasal spray in healthy volunteers
Source: Sci Rep. 2023 Sep 20;13:15648. doi: 10.1038/s41598-023-42539-7 (PMC10511465; doi:10.1038/s41598-023-42539-7)
Supplement: Supplementary file 5 — Supplementary Information 5. [file 41598_2023_42539_MOESM5_ESM.docx]

**Supplemental Methods for Biocompatibility Studies**

The biocompatibility studies comprised 5 tests as described below.

1. ***In vitro* cytotoxicity study by direct contact method**

The test and control items were used as such. Sterile filter paper measuring 2.5 cm^2^ (2.5 cm × 1 cm) was loaded with the NAS without any dilution and placed at the center of each culture flask covering approximately 10% of the culture area under sterile condition, as required by the guideline ISO 10993-5:2009. Control items, sterilized high density polyethylene (HDPE) (negative control) and natural rubber latex (positive control) measuring 2.5 cm^2^ (2.5 cm × 1 cm) were used as such and placed at the center of each culture flask. Triplicate cultures were set up for negative control, NAS and positive control. This fulfills the requirement of ISO 10993, Part 5:2009 and ISO 10993-12:2012 and ISO 10993-12:2021.

Exponentially growing Balb/c 3T3 cells were seeded in culture flasks at a concentration of 1×10^5^ cells per ml and maintained at 5% CO_2_ at 37 ± 1ºC, > 90% humidity for 24 h. On the day of treatment, after confirming confluency (80%) fresh medium was replaced in each culture flask. Then, the NAS, HDPE and natural rubber latex was placed at the center of each culture flask, covering approximately 10% of the culture area under sterile condition. Triplicate cultures were set up for negative control, NAS and positive control. After 24 h, qualitative evaluation was performed.

Qualitative evaluation

The cultures treated with the negative control did not show any cytotoxic response (grade 0) whereas the positive control showed a severe positive cytotoxic response (grade 4). The assay was therefore considered valid. No detectable zone around or under specimen was observed in the cultures treated with NAS (grade 0).

Quantitative evaluation

Viability greater than 70% was observed in the cultures treated with NAS, negative control. The positive control performed as expected.

1. **Skin sensitization study in guinea pigs**

The NAS was used at four different concentrations for the preliminary study (25% v/v, 50% v/v, 75% v/v in physiological saline and 100%). This fulfils the requirement of ISO 10993-12:2012 and ISO 10993-12:2021.

Initially, the range finding study (preliminary test) was conducted using four animals to determine the highest dose concentration that are well tolerated to cause only mild to moderate irritation and to determine the highest non-irritant dose of the NAS. Physiological saline was used as a negative control. The four different concentrations (25% v/v, 50% v/v, 75% v/v in physiological saline and 100%) of the NAS were applied (0.5 ml) as a topical patch at two different sites to all the four animals (one animal/concentration). Patches were held in contact with skin for 24 h by means of occlusive dressing. Skin reaction grading was performed using Magnusson and Kligman scale. After patch removal, no reaction or systemic effects were observed at 25% v/v, 50% v/v, 75% v/v and 100% in all the animals. Based on the results of the range finding study, 100% concentration (NAS as such without any dilution) was selected for the main study (topical induction and challenge phase) and based on the injectability, 100% concentration was selected for intradermal induction.

In main study, animals were divided into two groups; G1 - five guinea pigs for negative control, G2 - ten guinea pigs for NAS. The fur over the treatment sites were clipped and shaved on day of treatment. Induction of sensitization was a two-stage procedure with intradermal injections administered on day 0 (with FCA, vehicle and NAS). No local irritation (erythema & oedema) was found at test site B (NAS treated site) in the intradermal induction phase. Since no irritation was observed, pre-treatment with sodium lauryl sulphate was performed on day 6. On day 7, topical patch measuring 8 cm^2^ loaded with 0.5 ml of NAS and physiological saline was applied topically in the test and control groups respectively, on the same site as that of intradermal injections. The over patch was covered loosely with an occlusive dressing which was held in place for 48 h.

On day 21, challenge patch measuring 8 cm^2^ loaded with 0.5 ml of NAS and physiological saline was applied topically in the left and right flank of each animal for 24 h. Skin reaction grading was performed using Magnusson and Kligman scale at 24 h and 48 h, after removing the challenge patch according to ISO 10993-10:2021.

1. **Intracutaneous reactivity test in New Zealand white rabbits**

Since the NAS is a liquid, it was used as such without any dilution. Physiological saline was used as negative control. This fulfils the requirement of ISO 10993-12:2012 and ISO 10993-12:2021.

About 16 h and 21 minutes prior to intracutaneous injections, fur on all the rabbits were closely clipped off their backs, allowing sufficient distance on both sides of the spine for injection. NAS and negative control were injected intracutaneously (0.2 ml of injection at five test sites/treatment). Animals were observed at 24, 48, and 72 h for morbidity, mortality and abnormal clinical signs and symptoms after injection. The skin reactions were visually scored according to ISO 10993-23:2021, at 24 h, 48 h and 72 h post injection (Table S21). Observations were graded on a numerical scale for both the NAS and control.

Table S21 Grading score for the skin reactions after intracutaneous injections

| **Reaction** | **Numerical grading** |
| --- | --- |
| **Erythema and eschar formation** | |
| No erythema | 0 |
| Very slight erythema (barely perceptible) | 1 |
| Well-defined erythema | 2 |
| Moderate erythema | 3 |
| Severe erythema (beet-redness) to eschar formation preventing grading of erythema | 4 |
| **Oedema formation** | |
| No oedema | 0 |
| Very slight oedema (barely perceptible) | 1 |
| Well-defined oedema (edges of area well defined by definite raising) | 2 |
| Moderate oedema (raised approximately 1 mm) | 3 |
| Severe oedema (raised more than 1 mm extending beyond exposure area) | 4 |
| **Maximal possible score for irritation** | 8 |
| Source: ISO 10993- Part 23: 2021 |  |

After 72 h grading, all erythema and oedema grades at 24 h, 48 h and 72 h were totalled for each NAS or control for each individual animal. For calculating the score of a NAS and control on each individual animal, the derived value was divided each of the totals by 15 (3 scoring periods × 5 test or control sample injection sites). To determine the overall mean score for each NAS and each corresponding control, the scores for the 3 animals were added and divided by three.

The final NAS score was obtained by subtracting the score of the control from the NAS score. If the difference between the mean reaction grades (erythema/oedema) for the NAS and the control is greater than 1.0, then the NAS was considered to cause intracutaneous reactivity.

1. **Acute systemic toxicity test in swiss albino mice**

Since the NAS is a liquid, it was used as such without any dilution. Physiological saline was used as negative control. This fulfils the requirements of ISO 10993-12:2012 and ISO 10993-12:2021.

Two groups of mice, each comprising of five males were used for this study. Group 1 animals were treated orally using 1 ml syringe with negative control (physiological saline). Similarly, Group 2 animals were treated orally with NAS (as such) without any dilution. The details are given in the Table S22.

Table S22 The details of acute systemic toxicity study

| Group No. | No. of animals | Sample | Route of administration | Dose  Volume |
| --- | --- | --- | --- | --- |
| G1 | 5 | Physiological saline (Negative control) | Oral | 50 ml/kg b.w. |
| G2 | 5 | NAS | Oral | 50 ml/kg b.w. |

Animals were observed daily for mortality and morbidity throughout the experiment. Body weights of each animal were recorded prior to dosing, at 24 ± 2 h, 48 ± 2 h and 72 ± 2 h following NAS administration. Clinical observation was monitored at the time of NAS administration (0 h), then within 30 min and at 4 h, 24 ± 2 h, 48 ± 2 h and 72 ± 2 h following the NAS administration for any clinical signs of toxicity.

1. **Subacute (28-days) systemic toxicity study in Wistar rats**

According to OECD 407, a limit test dose of 1000 mg/kg/day was selected for this study. The equivalent weight of 1 ml of the NAS was determined and the volume was adjusted to obtain 1000 mg. This volume was made up to 10 ml with sterile physiological saline; animals in the test group were dosed at 10 ml/kg body weight.

All the animals were fasted overnight throughout the study period. The NAS was administered to rat daily once via oral route for a period of 28 days using oral gavage as shown in Table S23.

Table S23 The details of subacute (28-days) systemic toxicity study

| **Group No.** | **No. & Sex of animals** | **Dose** | **Route of Administration** | **Dose Volume** |
| --- | --- | --- | --- | --- |
| G1 – Negative control | 6 M + 6 F | Physiological saline | Oral | 10 ml/kg b.w * |
| G2 - NAS | 6 M + 6 F | NAS |  |  |
| * Administration of Substances to Laboratory Animals: Routes of Administration and Factors to Consider, J Am Assoc Lab Anim Sci. 2011; M-Male; F-Female. | | | | |

Animals were observed daily for mortality, morbidity and signs/symptoms of toxicity. Body weight and feed consumption were recorded weekly. At the end of the test period (28 days), blood samples were collected for haematological and biochemical analysis. Animals were then euthanized and necropsy was performed which includes careful examination of the external surface of the body, all orifices, and the cranial, thoracic and abdominal cavities and their contents was conducted. Organs were weighed for group comparison and processed for histopathology evaluation as per Tier I listed in Annex F of ISO 10993-11:2017. Clinical observations were made at least once in a day, throughout the study period, preferably at the same time of each day and recorded. Signs noted include, but not limited to the following observed signs as showed in Table S24.

Table S24 Clinical signs

| **Clinical Observation** | **Code No.** | **Observed Sign** |
| --- | --- | --- |
| Respiratory | 1 | Dyspnoea |
|  | 2 | Abdominal breathing |
|  | 3 | Gasping |
|  | 4 | Apnoea |
|  | 5 | Cyanosis |
|  | 6 | Tachypnea |
|  | 7 | Nostril discharges |
| Motor activities | 8 | Catatonia |
|  | 9 | Somnolence |
|  | 10 | Anaesthesia |
|  | 11 | Catalepsy |
|  | 12 | Ataxia |
|  | 13 | Unusual locomotion |
|  | 14 | Prostration |
|  | 15 | Tremors |
|  | 16 | Fasciculation |
| Convulsion | 17 | Clonic |
|  | 18 | Tonic |
|  | 19 | Tonic-Clonic |
|  | 20 | Asphyxial |
|  | 21 | Opisthotonos |
| Reflexes | 22 | Corneal |
|  | 23 | Righting |
|  | 24 | Myotact |
|  | 25 | Light |
|  | 26 | Startle reflex |
| Ocular signs | 27 | Lacrimation |
|  | 28 | Miosis |
|  | 29 | Mydriasis |
|  | 30 | Exophthalmos |
|  | 31 | Ptosis |
|  | 32 | Corneal opacity |
|  | 33 | Iritis |
|  | 34 | Conjunctivitis |
|  | 35 | Chromodacryorrhea |
|  | 36 | Relaxation of nictitating membrane |
| Cardiovascular signs | 37 | Bradycardia |
|  | 38 | Tachycardia |
|  | 39 | Arrhythmia |
|  | 40 | Vasodilation |
|  | 41 | Vasoconstriction |
| Salivation | 42 | Excessive |
| Piloerection | 43 | Rough hair |
| Analgesia | 44 | Decrease reaction |
| Muscle tone | 45 | Hypotonia |
|  | 46 | Hypertonia |
| Gastrointestinal | 47 | Constipation |
|  | 48 | Diarrhoea |
|  | 49 | Retching |
|  | 50 | Emesis |
| Urinary | 51 | Hematuria |
|  | 52 | Diuresis |
| Skin | 53 | Edema |
|  | 54 | Erythema |

**Hematology and Clinical Biochemistry**

At the end of experiment, the blood samples were collected (after anaesthesia) prior to humanely sacrificing the animals. The haematological examination includes:

1. Hematocrit

2. Clotting potential (PT & APTT)

3. Hemoglobin concentration

4. Red blood cell count

5. White blood cell count

6. WBC differential count

7. Platelet count

Clinical biochemistry determinations to investigate major toxic effects in tissues and, specifically, effects on kidney and liver, were performed on blood samples. Determinations in plasma or serum included the following:

1. Albumin

2. ALP

3. ALT

4. AST

5. Calcium

6. Chloride

7. Cholesterol

8. Creatinine

9. GGT

10. Glucose

11. Inorganic phosphorus

12. Potassium

13. Sodium

14. Total bilirubin

15. Total protein

16. Triglycerides

17. Urea nitrogen

Urinalysis was performed during the last week of the experiment using timed (16 h to 24 h) urine volume collection. The following parameters were analysed.

1. Appearance

2. Volume

3. Sediment

4. Urobilinogen

5. Bilirubin

6. Glucose

7. Ketones

8. Occult Blood

9. Protein

10. Nitrite

11. Leukocytes

12. pH

13. Specific gravity or osmolality

Since no NAS related clinical signs were observed throughout the study period, additional parameters including enzymes and immunoglobulin levels were not performed.

**Organ weight**

Liver, kidneys, adrenals, testes, epididymides, uterus, ovaries, thymus, spleen, brain (includes cerebrum, cerebellum and pons) and heart of all animals (apart from those found moribund and/or inter currently killed) were trimmed of any adherent tissue, as appropriate, and their wet weight was measured as soon as possible after dissection to avoid drying. Paired organs were weighed together. Relative weight of individual organ was calculated by dividing the absolute organ weight with the body weight of the animal and multiplying by 100. Organ weight of animals that are found dead during the study was not recorded.

**Histopathology**

The following list of organs/tissues (Tier II) were collected and preserved from all the animals as mentioned in Annex E of ISO 10993-11:2017.

1. Adrenals

2. All gross lesions (including treatment sites)

3. Aorta

4. Bone marrow (sternum)

5. Brain (including cerebrum, cerebellum and pons)

6. Caecum

7. Colon

8. Duodenum

9. Epididymis

10. Oesophagus

11. Eyes

12. Femur

13. Heart

14. Ileum

15. Jejunum

16. Kidneys

17. Liver

18. Lungs & bronchi

19. Lymph nodes (cervical and mesenteric)

20. Mammary gland (female)

21. Muscle (skeletal)

22. Nerve (sciatic)

23. Ovaries

24. Pancreas

25. Parathyroid

26. Pituitary

27. Prostate

28. Rectum

29. Salivary glands

30. Seminal vesicles

31. Skin

32. Spinal cord

33. Spleen

34. Stomach

35. Testes

36. Thymus

37. Thyroid

38. Trachea

39. Urinary Bladder

40. Uterus

41. Vagina

Limited histopathological analysis was conducted for below mentioned organs/tissues (Tier I) in the control and treated groups as listed in Annex F of ISO 10993-11:2017.

1. Heart

2. Liver

3. Adrenals

4. Kidneys

5. Skin

6. Spleen

7. Muscle

8. Brain

9. Testes/ Ovaries

10. Lungs and Bronchi

11. Femur

12. Bone marrow (sternum)
